# Toward Food Freshness Monitoring: Coordination Binding–Based Colorimetric Sensor Array for Sulfur-Containing Amino Acids

**DOI:** 10.3389/fchem.2021.685783

**Published:** 2021-06-17

**Authors:** Xiaojun Lyu, Wei Tang, Yui Sasaki, Jie Zhao, Tingting Zheng, Yang Tian, Tsuyoshi Minami

**Affiliations:** ^1^ Institute of Industrial Science, The University of Tokyo, Tokyo, Japan; ^2^ Key Laboratory of Green Chemistry and Chemical Processes, Department of Chemistry, School of Chemistry and Molecular Engineering, East China Normal University, Shanghai, China

**Keywords:** glutathione, cysteine, chemosensor array, food analysis, regression analysis, colorimetric sensing

## Abstract

Herein, a self-assembled colorimetric chemosensor array composed of off-the-shelf catechol dyes and a metal ion (i.e., Zn^2+^) has been used for the sulfur-containing amino acids (SCAAs; i.e., glutathione, glutathione disulfide, L–cysteine, DL–homocysteine, and L–cystine). The coordination binding–based chemosensor array (CBSA) fabricated by a competitive assay among SCAAs, Zn^2+^ ions, and catechol dyes [i.e., pyrocatechol violet (PV), bromopyrogallol red (BPR), pyrogallol red (PR), and alizarin red S (ARS)] yielded fingerprint-like colorimetric changes. We succeeded in the qualification of SCAAs based on pattern recognition [i.e., a linear discrimination analysis (LDA)] with 100% correct classification accuracy. The semiquantification of reduced/oxidized forms of SCAAs was also performed based on LDA. Furthermore, we carried out a spike test of glutathione in food samples using the proposed chemosensor array with regression analysis. It is worth mentioning that we achieved a 91–110% recovery rate in real sample tests, which confirmed the accuracy of the constructed model. Thus, this study represents a step forward in assessing food freshness based on supramolecular analytical methods.

## Introduction

To date, there is an increasing demand from consumers to evaluate the safety of food products. Freshness is the main standard for food quality assessment, including the physical form (Luo et al., 2021), the number of microorganisms (Jacxsens et al., 2003), and biochemical components (Lonchamp et al., 2009). Among the common biochemical components, sulfur-containing amino acids (SCAAs) including L–cysteine (Cys) (Cebi et al., 2017; Garcia et al., 2015), L–cystine (CySS) (Chen and Li, 2019), DL–homocysteine (HCys) (Hoey et al., 2007), glutathione reduced form (GSH) (Xu et al., 2015), and glutathione oxidized form (GSSG) (Moreira et al., 2011) have been used as analyte markers in food samples, including wine (Valero et al., 2003), wheat flour (Reinbold et al., 2008), and fruit juice (Fracassetti et al., 2011), to evaluate food quality. The reduced form of SCAAs acts as antioxidants in food (Nikolantonaki et al., 2018), which is oxidized by air over time. Thus, by quantifying the reduced form and the oxidized form of SCAAs, the freshness of food samples can be assessed.

SCAAs are currently quantified by instrumental methods (i.e., high-performance liquid chromatography (HPLC) (Zhu et al., 2020) and mass spectrometry (MS) (Kuster et al., 2008), or enzyme-linked immunosorbent assay (ELISA) (Kurose et al., 1997) because of their high reliability and accuracy. However, the requirement of expensive instruments, complex sensing procedures, and trained personnel limits their application for rapid and straightforward analysis. To simplify the sensing procedures, chemosensors have become a promising option, which can exhibit optical property changes based on molecular recognition (Hyman and Franz, 2012; Lee et al., 2015; Wu et al., 2015; Yan et al., 2017; Khorasani et al., 2019; Roy, 2021). In this regard, several optical chemosensors have been developed for natural amino acids (Buryak and Severin, 2005; Ma et al., 2013; Yin et al., 2013; Sener et al., 2014; Ghasemi et al., 2015; Meng et al., 2015; Chao and Zhang, 2017; Gholami et al., 2019; Liu et al., 2019; Xu et al., 2020). However, these chemosensors require complicated synthetic processes (Yang et al., 2011; Song et al., 2016; Yang et al., 2018), which limit their practical usage in real-world scenarios. Chemosensor arrays by supramolecular interactions with pattern recognition can avoid the synthetic processes and simultaneously quantify multiple analytes (Cao et al., 2020; Sasaki et al., 2021a; Sasaki et al., 2021b; Lyu et al., 2021). To the best of our knowledge, the development of a colorimetric sensor array using only a combination of off-the-shelf reagents for simultaneous SCAA detection has not yet been reported.

**FIGURE 1:**
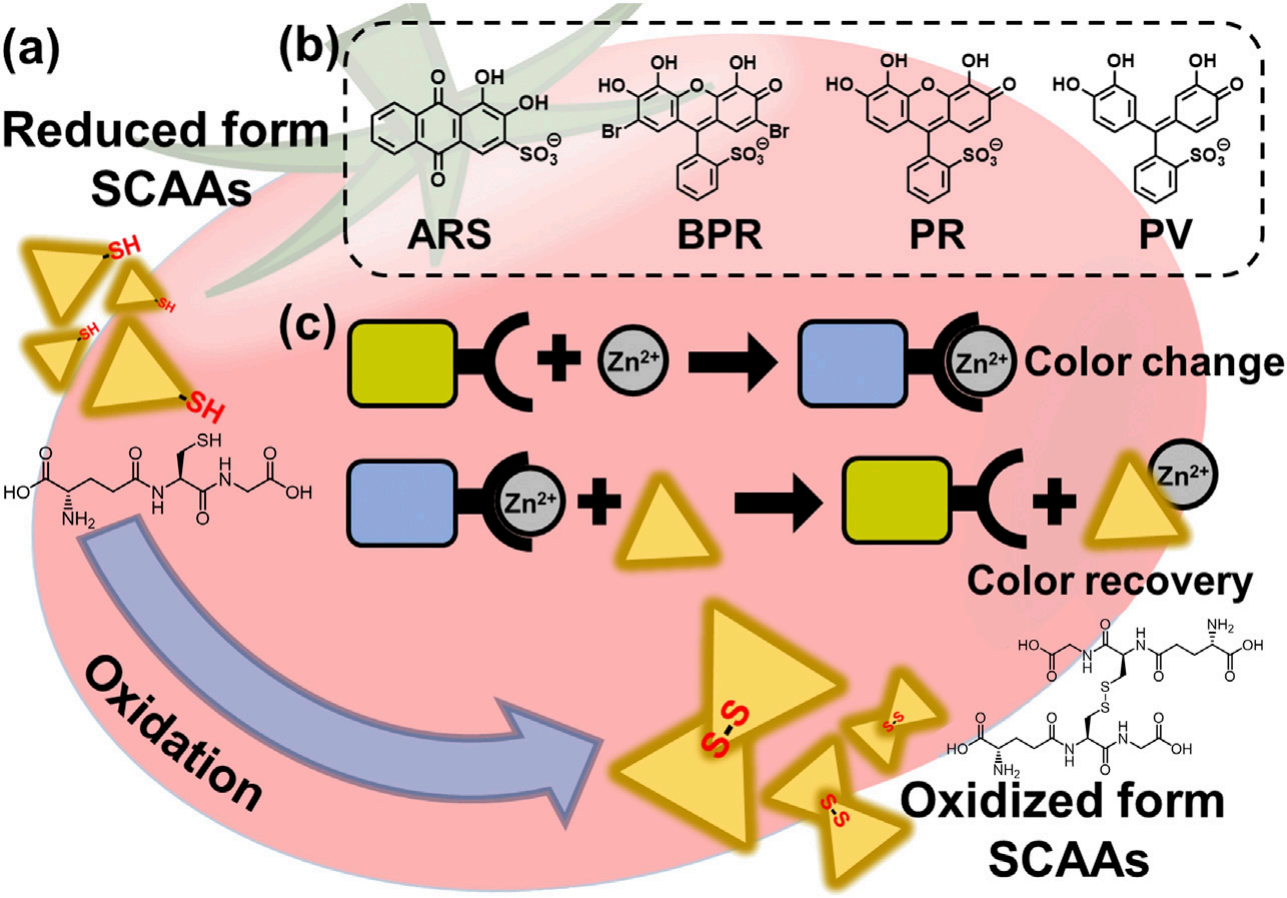
**(A)** Schematic illustration of the SCAA oxidation (e.g., glutathione). **(B)** Chemical structure of catechol dyes (PV, BPR, PR, and ARS). **(C)** Illustrated detection mechanism of SCAAs.

Herein, we report a simple, rapid, and accurate coordination binding–based chemosensor array (CBSA) for the high-throughput colorimetric detection of SCAAs (GSH, GSSG, Cys, hCys, and CySS). Four catechol dyes [i.e., pyrocatechol violet (PV), bromopyrogallol red (BPR), pyrogallol red (PR), and alizarin red S (ARS)] were employed as colorimetric indicators (Sasaki et al., 2019), and Zn^2+^ ions were used as the color and binding manipulator (Kaushik et al., 2015). The catechol dyes produce the dye–Zn^2+^ coordination complex upon the addition of Zn^2+^ ions, which exhibit colorimetric changes (Hamedpour et al., 2019). Subsequently, colorimetric changes can be observed by the addition of SCAAs because of the generation of coordination complexes of Zn^2+^ and SCAAs, which dissociate the dye–Zn^2+^ complex with a colorimetric recovery (Figure 1). The use of commercially available reagents avoids complex synthesis, which is a major advantage for the simple establishment of a sensor array (Sasaki et al., 2021c). In this study, various cross-reactive colorimetric responses demonstrated by the CBSA were analyzed using chemometric methods, including a linear discrimination analysis (LDA) (Anzenbacher et al., 2010) and regression analysis (SVM) (Hamel, 2009; Minami et al., 2012). The LDA is a mathematical method used in statistics to establish a linear combination to characterize two or more groups of objects, which could be used for qualitative and semiquantitative analyses of the targets. The SVM is a supervised learning model for data classification and regression analysis. To predict the unknown concentrations of the target samples, we established the SVM model with the calibration dataset using standard solutions. Notably, we quantified the pseudo-oxidation processes of SCAAs in aqueous media and predicted the GSH concentration in tomato and grapefruit juice (Minich and Brown, 2019). These results indicate that our simple preparation and user-friendly sensing system could achieve high-throughput analysis for SCAAs, which would be a step forward for assessing food freshness based on supramolecular analytical methods.

## Materials and Methods

### Materials

SCAAs (Cys, CySS, GSH, and HCys), 1,4-benzoquinone (pBQ), 3-mercaptopropionic acid (3-MPA), PR, BPR, PV, and other amino acids [L–alanine (Ala), L–arginine (Arg), L–aspartic acid (Asp), L–glutamine (Gln), L–glutamic acid (Gln), glycine (Gly), L–histidine (His), L–isoleucine (Ile), L–leucine (Leu), L–lysine (Lys), L–methionine (Met), L–phenylalanine (Phe), L–proline (Pro), L–serine (Ser), L–threonine (Thr), L–tryptophan (Trp), L–tyrosine (Tyr), and L–valine (Val)] were purchased from Tokyo Chemical Industries Co. Inc. (Tokyo, Japan). Additionally, ARS, sodium chloride, methanol, and zinc nitrate hexahydrate were purchased from FUJIFILM Wako Pure Chemical Co. Inc. (Osaka, Japan). *N*-(2-hydroxyethyl)-1-piperazineethanesulfonic acid (HEPES) was purchased from Dojindo Laboratories (Kumamoto, Japan). All chemicals were used without further purification. All aqueous samples were prepared with Milli-Q water (18.2 MΩcm) (Millipore, Bedford, MA, United States).

### Measurements

The UV-Vis spectra were measured within the wavelength range of 350–800 nm at a scan rate of 240 nm/min using a Shimadzu UV-2600 UV-Vis spectrophotometer. The dye (40 μM) was mixed with Zn^2+^ solutions at various concentrations and incubated at room temperature (25°C) for 10–60 min (i.e., ARS, BPR, and PR for 60 min, and PV for 10 min) in HEPES buffer (50 mM) with 10 mM NaCl at pH 7.4. Titration isotherms were prepared from the variations with maximum absorption at 615 nm for PV, 557 nm for BPR, 543 nm for PR, and 515 nm for ARS, respectively.

Qualitative and quantitative analyses were carried out by array experiments in 384-well microplates using a microplate reader (SYNERGY H1, Biotek, Winooski, United States). For the array experiments, the dye–Zn^2+^ complex solution (90 μL) was first added to each well. Subsequently, the analyte and buffer solutions (10 μL) were mixed with the sensor solution. The microplate was then shaken for 3 min for incubation. Furthermore, a spike test using food samples was performed to evaluate the accuracy of the chemosensor array for real-world applications. A commercial tomato juice (Ito En tomato juice) was centrifuged at 14,000 rpm for 30 min to remove any insoluble matter. The supernatant fluid was diluted 40-fold and applied to a 384-well microplate without any further treatment. A series of standard GSH samples was calibrated with a concentration of 0–2 mM.

LDA was applied for qualitative analysis based on the raw dataset without any further treatment using SYSTAT 13. Moreover, a semiquantitative assay of the mixture of GSH/GSSG and Cys/CySS was also carried out using LDA. Student’s *t*-test was used to eliminate outlier data points. The quantitative analyses and real sample tests were conducted by a regression analysis based on a supporting vector machine (SVM) with Solo 7.5.2. Two parameters [root-mean-square errors for calibration (RMSEC) and prediction (RMSEP)] were applied to confirm the accuracy of the constructed models.

## Discussions

**FIGURE 2:**
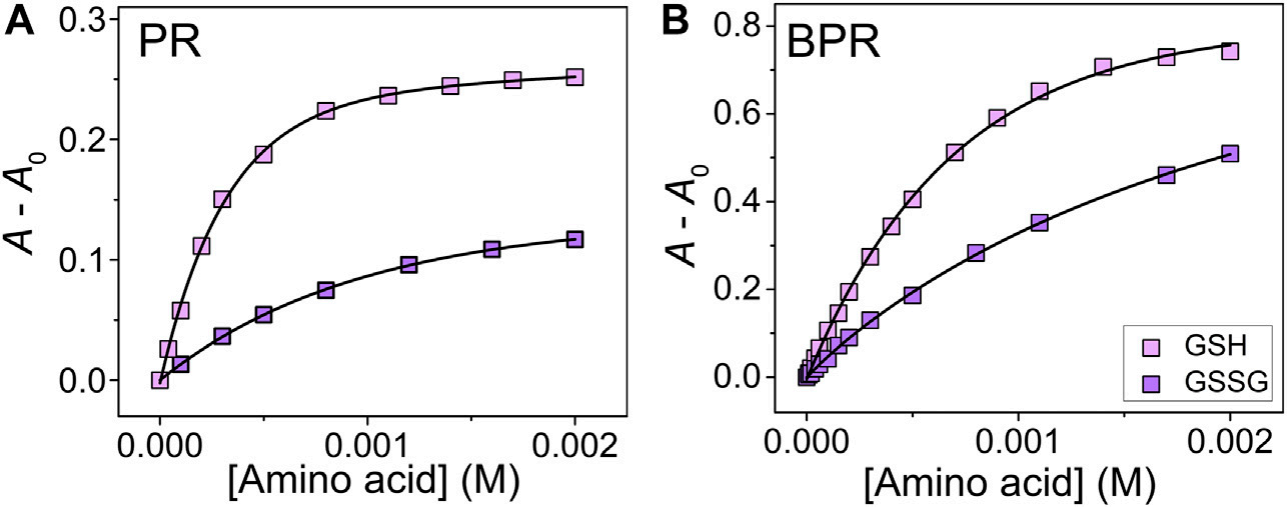
UV-Vis titration isotherm of **(A)** PR–Zn^2+^ and **(B)** BPR–Zn^2+^ to GSH and GSSG (0–2.0 mM) at 25°C. The concentrations of dye and Zn^2+^ were 40 μM.

The complexation of catechol dyes and Zn^2+^ ions in a HEPES buffer (50 mM) with NaCl (10 mM) at pH 7.4 and at 25°C was evaluated according to a previous study (Hamedpour et al., 2019). To maintain the ionic strength, sodium chloride (10 mM) was added. Each dye complex showed specific colorimetric changes upon the addition of SCAAs (GSH, GSSG, Cys, and hCys) (see Supplementary Material). The spectral shift accompanying the color recovery was observed by the ascension of the concentration, indicating the decomposition of the Zn^2^–catechol dye complex. Because of the low solubility of CySS in the buffer solution, we could not apply UV-Vis titration for CySS in this study. Association constants (*K*_assoc_) for the SCAAs with Zn^2+^ ions were calculated by titration isotherms according to a nonlinear regression fitting method (see supporting material) (Hargrove et al., 2010). The *K*_assoc_ for GSH were determined as follows: PV: (4.7 ± 0.3) × 10^3^ M^−1^; BPR: (2.8 ± 0.8) × 10^4^ M^−1^; PR: (1.5 ± 0.3) × 10^5^ M^−1^; and ARS: (6.5 ± 0.6) × 10^5^ M^−1^. In the case of GSSG, the *K*_assoc_ were calculated as (1.6 ± 0.1) × 10^4^ M^−1^, (9.6 ± 1.8) × 10^3^ M^−1^, (2.4 ± 0.6) × 10^4^ M^−1^, and (9.2 ± 1.7) × 10^6^ M^−1^ PV for, BPR, PR, and ARS, respectively. With the various binding affinities for the targets, our proposed chemosensor array could be applied for simultaneous qualitative and quantitative SCAA detection. Interestingly, the reduced/oxidized form of glutathione showed different isotherms toward the dye–Zn^2+^ complex (Figure 2), which indicates that we could monitor the oxidizing process of SCAAs based on colorimetric changes.

Subsequently, we applied natural amino acids to our proposed chemosensor array for the selectivity test. Most of the natural amino acids [i.e., valine (Val), tyrosine (Tyr), tryptophan (Trp), threonine (Thr), serine (Ser), proline (Pro), phenylalanine (Phe), methionine (Met), lysine (Lys), leucine (Leu), isoleucine (Ile), glycine (Gly), glutamic acid (Glu), glutamine (Gln), aspartic acid (Asp), asparagine (Asn), arginine (Arg), and alanine (Ala)] showed slight or almost no response, whereas histidine (His) caused significant absorbance changes with the peak wavelength shifts. This response was due to the high association constant of Zn^2+^ ions and His compared to the aforementioned natural amino acids (Krężel and Maret, 2016). In addition, the selectivity test indicated that the sulfur group contributes to the sensing mechanism based on CBSA (see Supplementary Material) (Namuswe and Berg, 2012). Thus, we attempted to apply a high-throughput assay to the aforementioned five analytes (i.e., GSH, GSSG, hCys, Cys, and His). We selected LDA among the pattern recognition methods because it can reduce the dimensionality and evaluate classification accuracy based on a leave-one-out cross-validation protocol (i.e., the jackknife method). The classification of the six cluster groups (one control group and five analyte groups) achieved 100% accuracy (Supplementary Figure S24). Thus, we can conclude that the proposed chemosensor array can discriminate between similar structural amino acids and reduced/oxidized forms of SCAAs.

**FIGURE 3:**
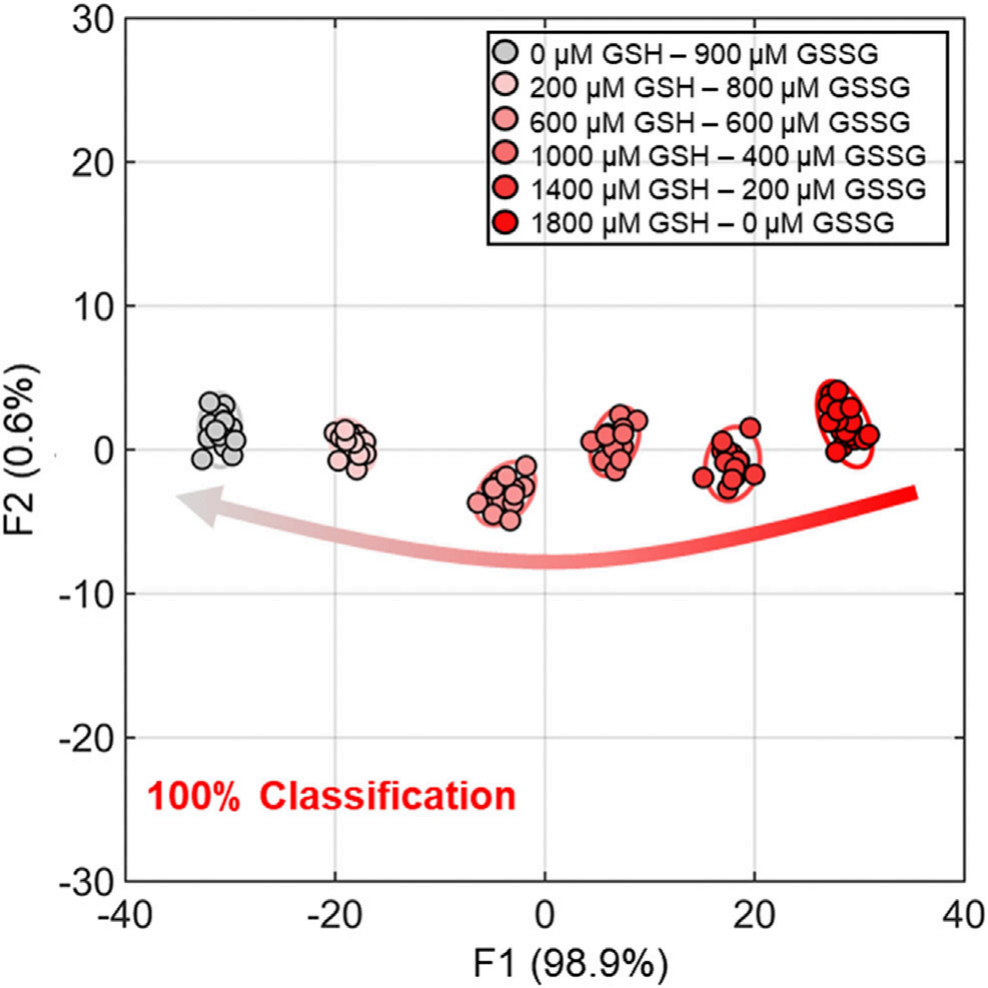
Semiquantitative LDA result for dynamic molar ratios of GSH and GSSG. The measurements were performed with eighteen repetitions for each concentration group for 100% classification accuracy. The confidence ellipses indicate 95% confidence rate.

**FIGURE 4:**
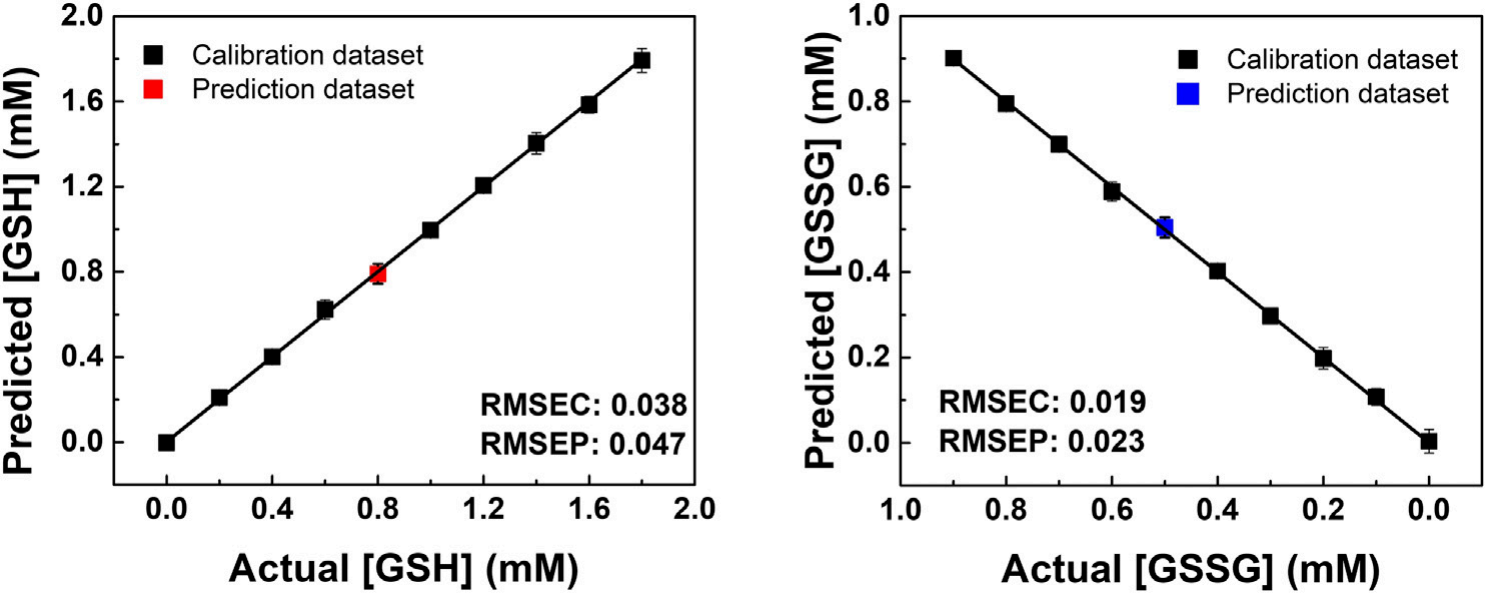
SVM regression analysis for quantitative analysis of the GSH and GSSG mixtures in various concentration ratios. The RMSEC and RMSEP values (shown as insets) represent the accuracy of the constructed model and the prediction.

In addition, semiquantitative LDA was carried out using the proposed chemosensor array for the mixture of oxidized/reduced SCAAs (i.e., glutathione and cysteine). For example, GSH–GSSG was selected as the representative analyte pair at various concentration ratios (Figure 3). The dynamic concentration changing rate of GSH and GSSG was selected as 2:1 to mimic the natural oxidizing process of glutathione (Supplementary Table S5). It is worth mentioning that the semiquantitative LDA of the mixture of oxidized/reduced SCAAs achieved a 100% correct classification rate. Furthermore, quantitative analyses were employed without sample preprocessing to investigate their capability for practical applications. Quantitative analyses were performed in a mixture containing GSH–GSSG or Cys–CySS in different molar ratios. An SVM-based regression analysis was carried out to establish a rapid and accurate assay (Figure 4). The SVM process includes two steps: the first step for the calibration of the measured data set and the second step for feature prediction of the unknown samples. More importantly, the limits of detection (LoDs) based on the 3σ method (Miller and Miller, 2018) were calculated as follows: 2.4 ppm for Cys, 2.0 ppm for hCys, 0.4 ppm for GSH, and 2.6 ppm for GSSG. As aforementioned, the constructed SVM model demonstrated the prediction of unknown concentrations of SCAAs with high accuracy, which indicates our array system could be applied to real sample analyses.

**FIGURE 5:**
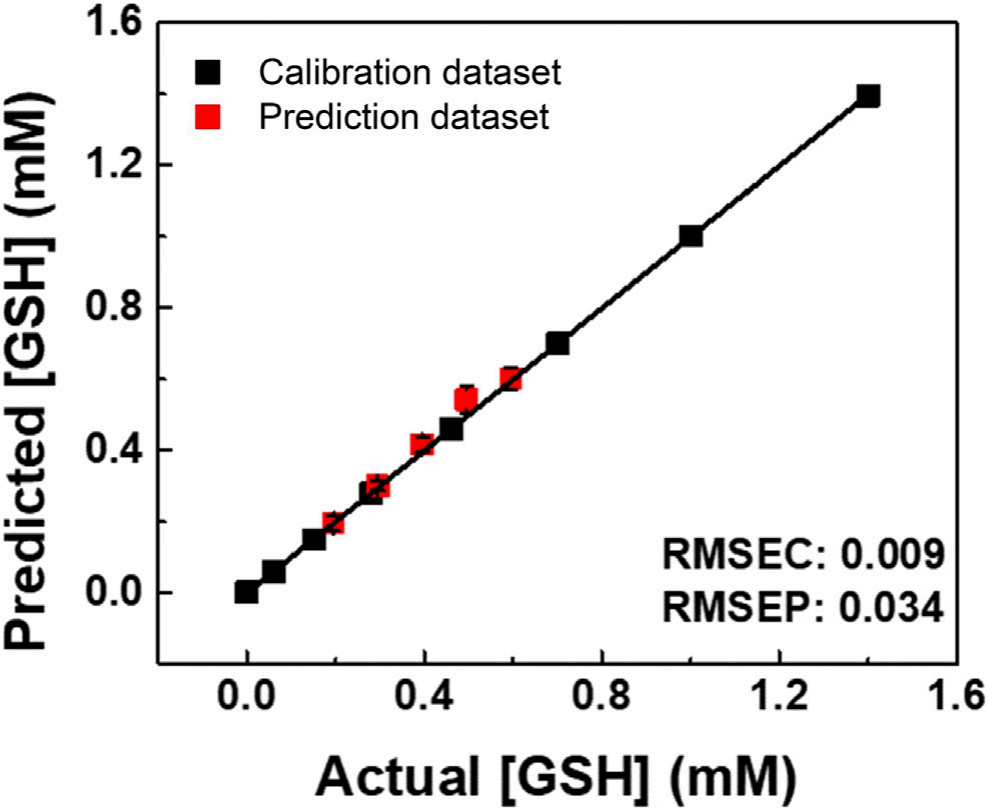
SVM regression for real sample analysis of GSH in a juice (Ito En tomato juice). The RMSEC and RMSEP values (shown as insets) represent the accuracy of the constructed model and the prediction.

**FIGURE 6:**
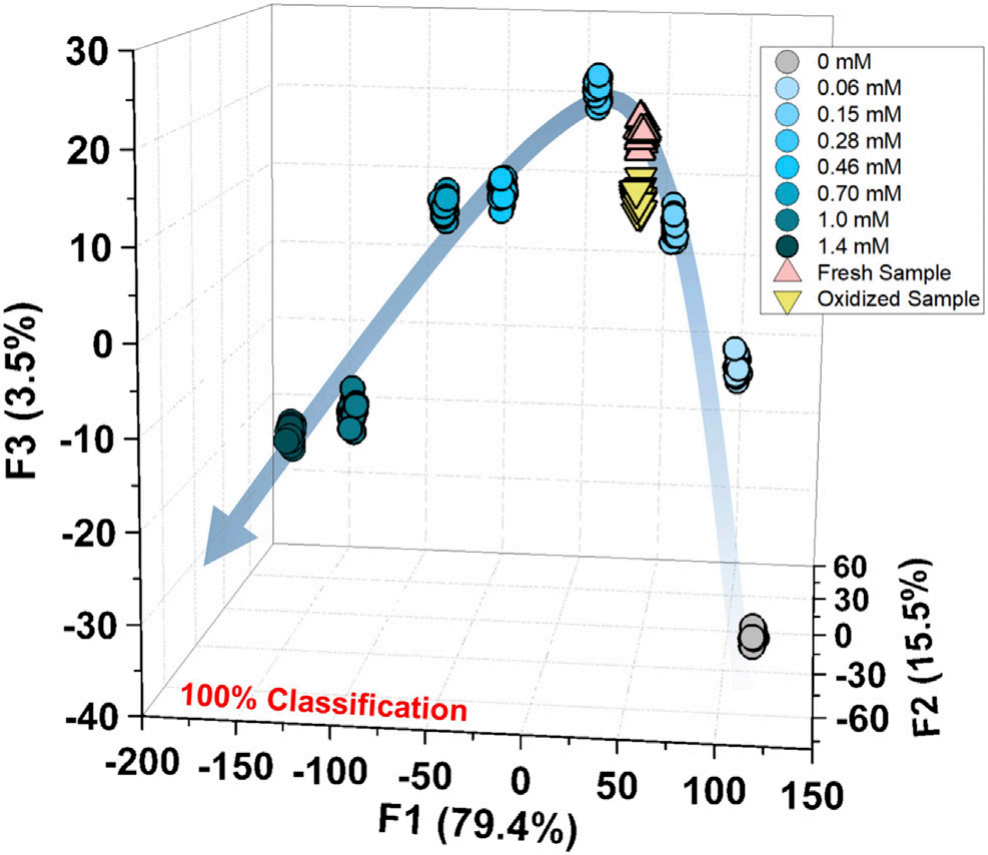
Semiquantitative analysis for the fresh and oxidized tomato samples with the calibration of GSH (0–1.4 mM). The measurements were repeated twenty times, resulting in 100% accurate classification.

Finally, we performed a spike test with GSH using concentrated tomato juice, grapefruit juice, and fresh tomato samples (Minich and Brown, 2019). Different concentrations of GSH were mixed with the diluted tomato juice and chemosensors, which were estimated by the previously calibrated SVM model (Figure 5). As shown in the Supplementary Table S6, the prediction of the GSH concentration in the real sample was successfully performed with a recovery rate of 91–110%. Moreover, the semiquantitative analysis offered 100% accurate classification of the fresh and oxidized tomato samples (Figure 6). The decrease in GSH concentration in the oxidized tomato sample was confirmed by HPLC–electrospray ionization MS (ESI-MS, see Supplementary Figures S32, S33). The LDA results indicated that the oxidized tomato sample demonstrated a closer distance to the low GSH concentration group, rather than the fresh sample (Figure 6). The result of the semiquantitative analysis and the spike test indicated that the chemosensor array combined with pattern recognition could be applied for quantifying unknown GSH concentrations, which suggests that the proposed method would become a rapid and promising method for the detection of SCAAs in food samples.

## Conclusion

In summary, we proposed a self-assembled colorimetric chemosensor array system for the qualitative and quantitative detection of SCAAs. The proposed array system was fabricated by CBSA utilizing off-the-shelf reagents, which avoided complicated synthetic processes. The reversible coordination binding of dye–Zn^2+^ complexes offered significant color changes upon the addition of the analytes. The LDA results reflected a clear classification of six groups (i.e., control, non-SCAAs, and reduced/oxidized SCAAs) with 100% classification accuracy. Moreover, quantitative analyses with high accuracy were achieved by the SVM, which allowed for the prediction of reduced/oxidized SCAAs in the mixtures. Most importantly, the spike test of GSH was performed in juice samples with high recovery rates. This study would lead to the application of supramolecular chemosensors for food freshness monitoring in the general society.

## Data Availability

The original contributions presented in the study are included in the article/Supplementary Material, further inquiries can be directed to the corresponding author.
