# Supplementary material for: Toward Food Freshness Monitoring: Coordination Binding–Based Colorimetric Sensor Array for Sulfur-Containing Amino Acids
Source: Front Chem. 2021 Jun 17;9:685783. doi: 10.3389/fchem.2021.685783 (PMC8248799; doi:10.3389/fchem.2021.685783)
Supplement: Supplementary file 1 [file DataSheet1.pdf]

Supplementary Material

Toward Food Freshness Monitoring: Coordination Binding–Based Colorimetric Sensor Array for Sulfur–Containing Amino Acids

Xiaojun Lyu[1], Wei Tang[1], Yui Sasaki[1], Jie Zhao[2], Tingting Zheng[2], Yang Tian[2] and Tsuyoshi Minami[1*]

[1]Institute of Industrial Science, The University of Tokyo, Tokyo, Japan

[2] Key Laboratory of Green Chemistry and Chemical Processes, Department of Chemistry, School of Chemistry and Molecular Engineering, East China Normal University, Shanghai, China

*** Correspondence:**
Tsuyoshi Minami
tminami@iis.u-tokyo.ac.jp

Contents

1. **UV-Vis titrations** **S2**
2. **Results of selectivity test** **S9**
3. **Results of quantitative analysis** **S10**
4. **Results of semiquantitative analysis** **S12**
5. **Results of quantitative analysis** **S15**
6. **Real-sample analysis for sufur–containing amino acids** **S20**
7. **HPLC analysis** **S20**
8. **LDA for the freshness monitoring of tomato** **S22**

Reference **S23**

1. UV-Vis titrations

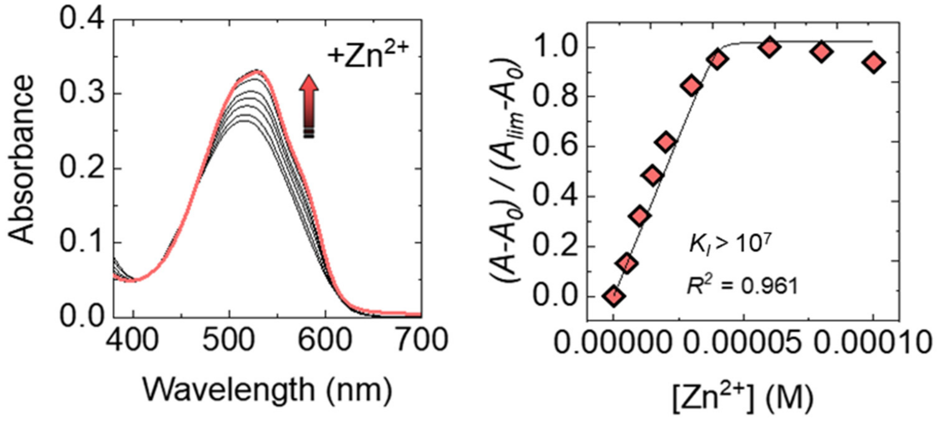

Supplementary Figure 1. UV-Vis spectra of ARS (40 μM) upon the addition of Zn^{2+} (0–100 μM) in a HEPES buffer solution (50 mM) with 10 mM NaCl at pH 7.4 (25 °C).

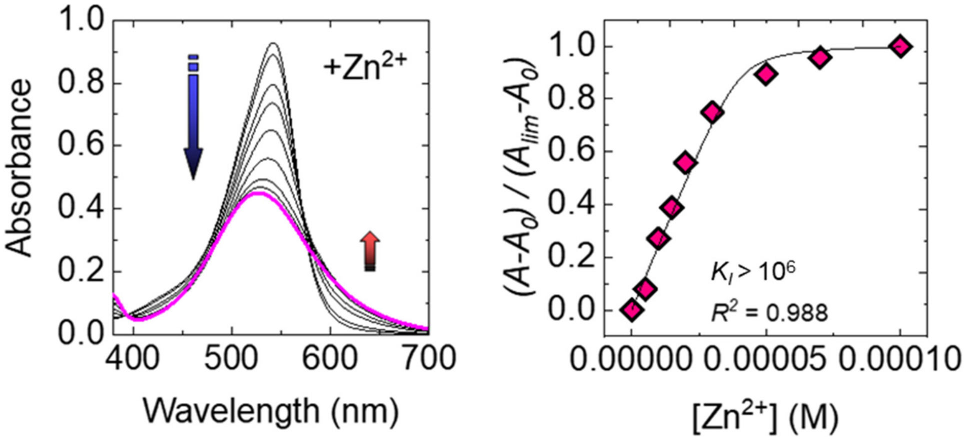

Supplementary Figure 2. UV-Vis spectra of BPR (40 μM) upon the addition of Zn^{2+} (0–100 μM) in a HEPES buffer solution (50 mM) with 10 mM NaCl at pH 7.4 (25 °C).

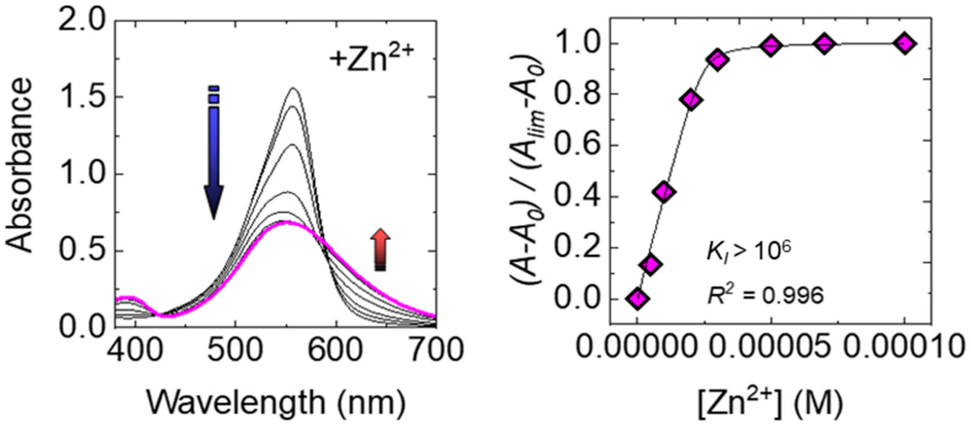

Supplementary Figure 3. UV-Vis spectra of PR (40 μM) upon the addition of Zn^{2+} (0–100 μM) in a HEPES buffer solution (50 mM) with 10 mM NaCl at pH 7.4 (25 °C).

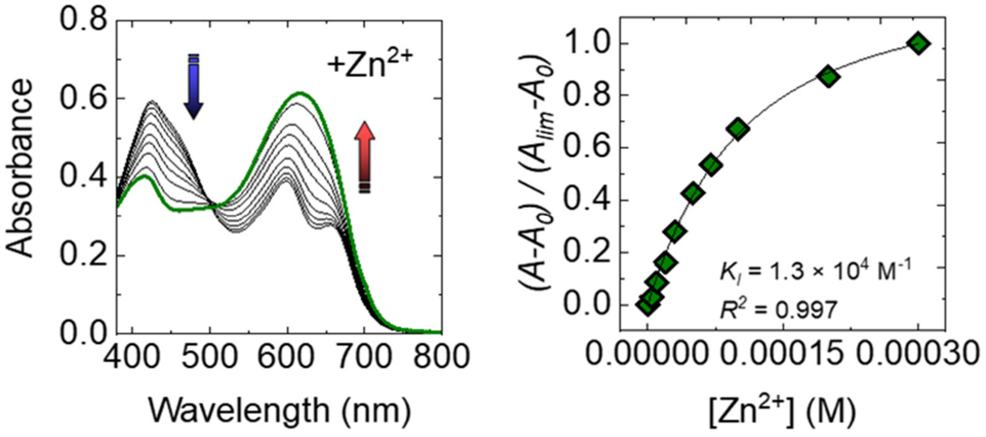

Supplementary Figure 4. UV-Vis spectra of PV (40 μM) upon the addition of Zn^{2+} (0–300 μM) complex in a HEPES buffer solution (50 mM) with 10 mM NaCl at pH 7.4 (25 °C).

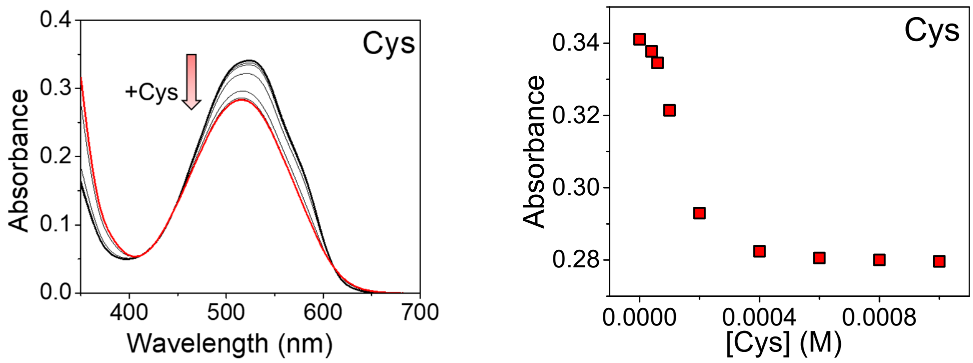

Supplementary Figure 5. UV-Vis spectra of the ARS (40 μM) – Zn^{2+} (40 μM) complex upon the addition of Cys (0–1.0 mM) in a HEPEs buffer solution (50 mM) with 10 mM NaCl at pH 7.4 (25 °C).

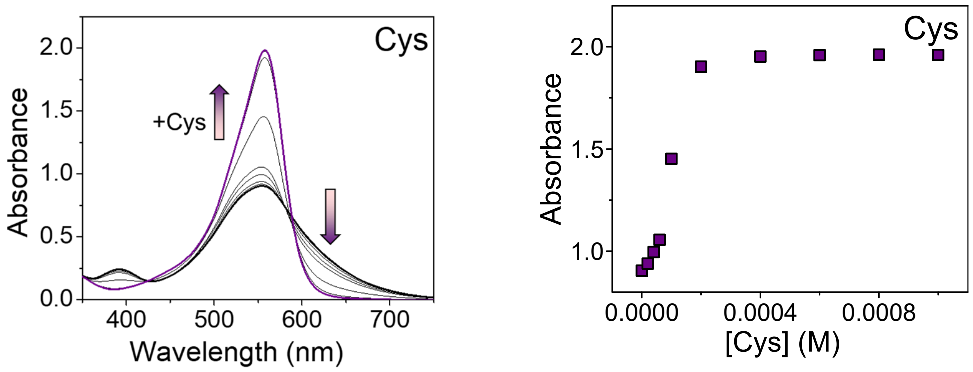

Supplementary Figure 6. UV-Vis spectra of the BPR (40 μM) – Zn^{2+} (40 μM) complex upon the addition of Cys (0–1.0 mM) in a HEPES buffer solution (50 mM) with 10 mM NaCl at pH 7.4 (25 °C).

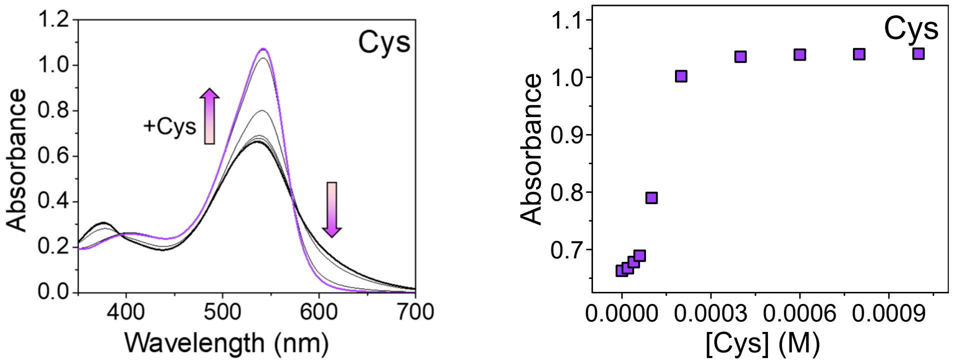

Supplementary Figure 7. UV-Vis spectra of the PR (40 µM) – Zn^{2+} (40 µM) complex upon the addition of Cys (0–1.0 mM) in a HEPES buffer solution (50 mM) with 10 mM NaCl at pH 7.4 (25 °C).

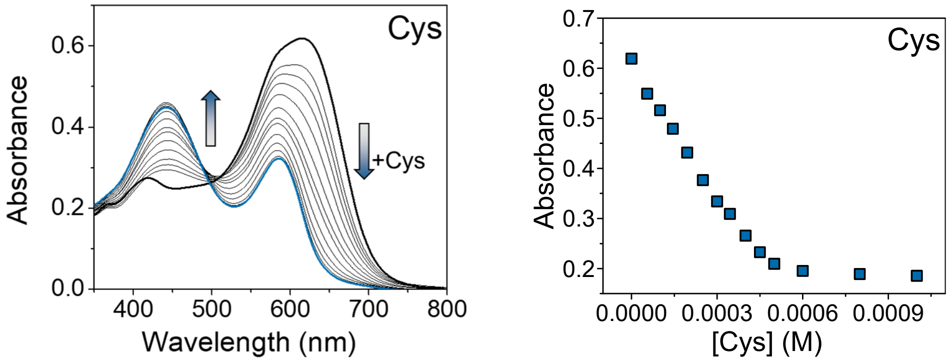

Supplementary Figure 8. UV-Vis spectra of the PV (40 µM) – Zn^{2+} (200 µM) complex upon the addition of Cys (0–1.0 mM) in a HEPES buffer solution (50 mM) with 10 mM NaCl at pH 7.4 (25 °C).

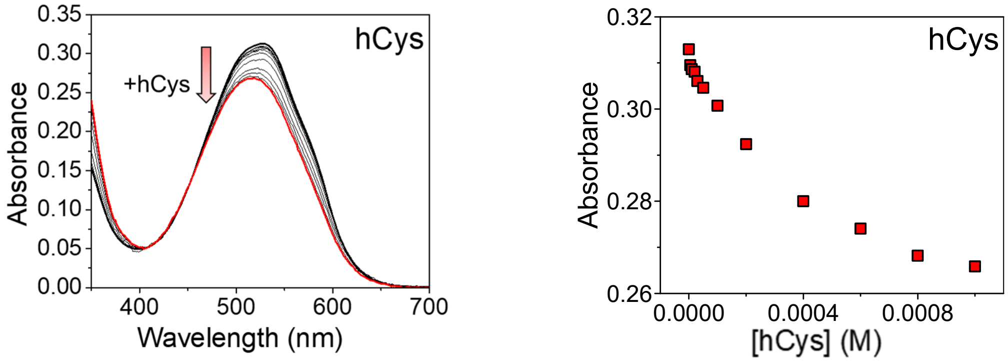

Supplementary Figure 9. UV-Vis spectra of the ARS (40 µM) – Zn^{2+} (40 µM) complex upon the addition of hCys (0–1.0 mM) in a HEPES buffer solution (50 mM) with 10 mM NaCl at pH 7.4 (25 °C).

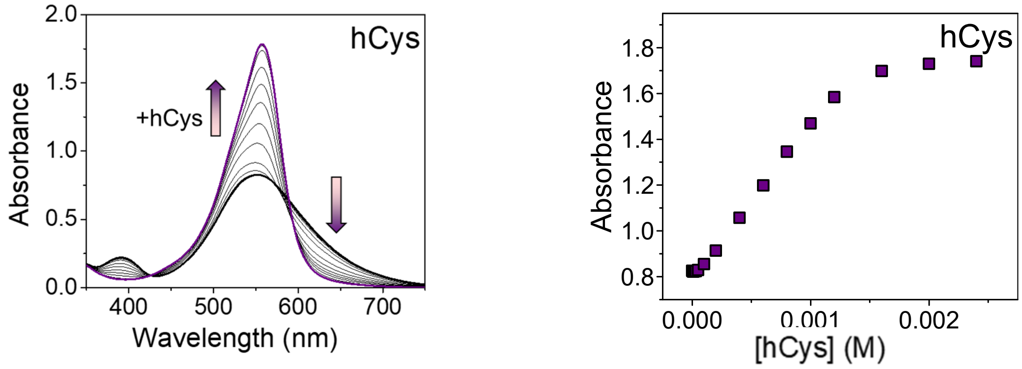

Supplementary Figure 10. UV-Vis spectra of the BPR (40 μM) – Zn^{2+} (40 μM) complex upon the addition of hCys (0–2.5 mM) in a HEPES buffer solution (50 mM) with 10 mM NaCl at pH 7.4 (25 °C).

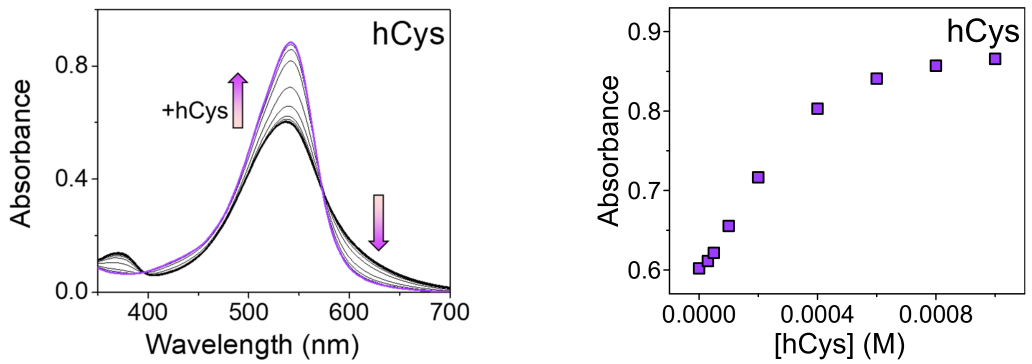

Supplementary Figure 11. UV-Vis spectra of the PR (40 μM) – Zn^{2+} (40 μM) complex upon the addition of hCys (0–1.0 mM) in a HEPES buffer solution (50 mM) with 10 mM NaCl at pH 7.4 (25 °C).

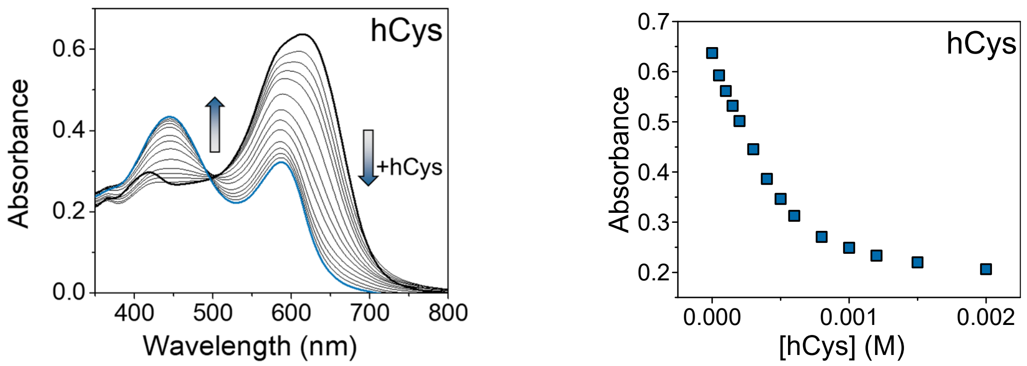

Supplementary Figure 12. UV-Vis spectra of the PV (40 μM) – Zn^{2+} (200 μM) complex upon the addition of hCys (0–2.0 mM) in a HEPES buffer solution (50 mM) with 10 mM NaCl at pH 7.4 (25 °C).

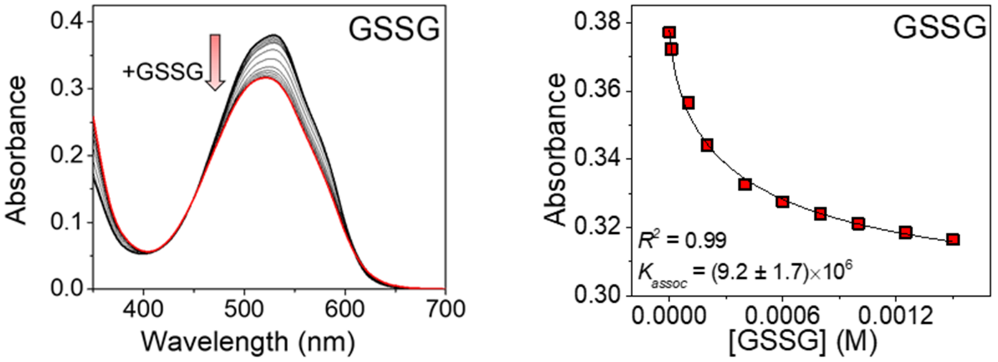

Supplementary Figure 13. UV-Vis spectra of the ARS (40 μM) – Zn^{2+} (40 μM) complex upon the addition of GSSG (0–1.5 mM) in a HEPES buffer solution (50 mM) with 10 mM NaCl at pH 7.4 (25 °C).

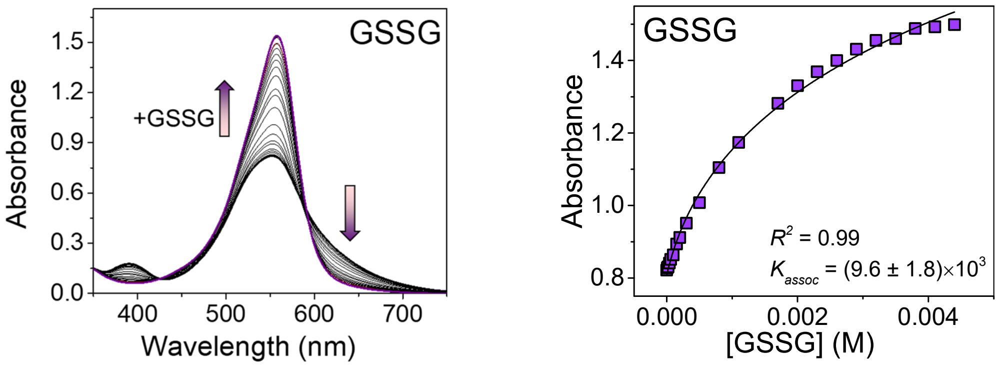

Supplementary Figure 14. UV-Vis spectra of the BPR (40 μM) – Zn^{2+} (40 μM) complex upon the addition of GSSG (0–4.0 mM) in a HEPES buffer solution (50 mM) with 10 mM NaCl at pH 7.4 (25 °C).

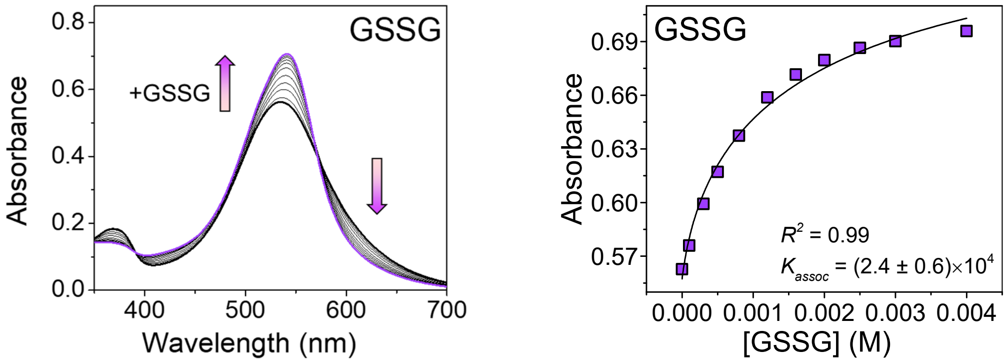

Supplementary Figure 15. UV-Vis spectra of the PR (40 μM) – Zn^{2+} (40 μM) complex upon the addition of GSSG (0–4.0 mM) in a HEPES buffer solution (50 mM) with 10 mM NaCl at pH 7.4 (25 °C).

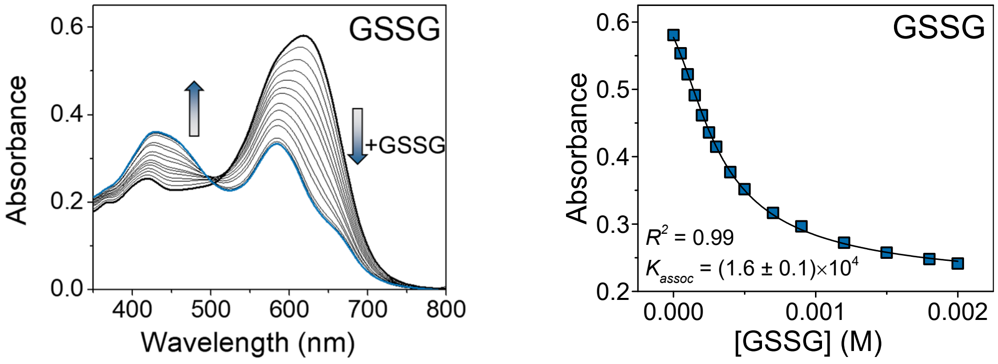

Supplementary Figure 16. UV–Vis spectra of the PV (40 μM) – Zn^{2+} (200 μM) complex upon the addition of GSSG (0–2.0 mM) in a HEPES buffer solution (50 mM) with 10 mM NaCl at pH 7.4 (25 °C).

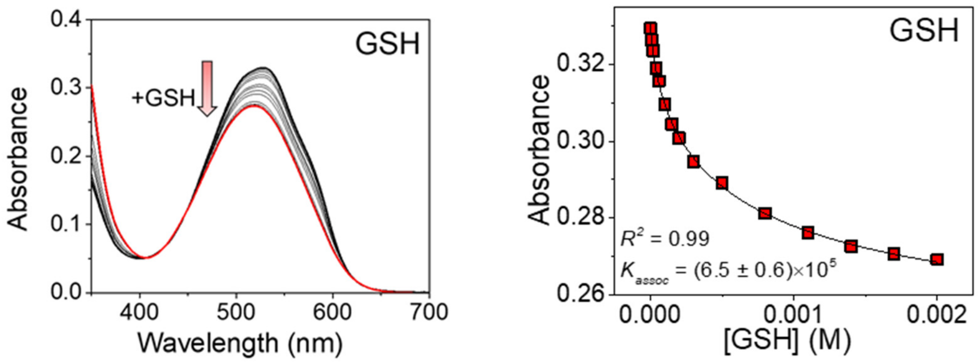

Supplementary Figure 17. UV–Vis spectra of the ARS (40 μM) – Zn^{2+} (40 μM) complex upon the addition of GSH (0–2.0 mM) in a HEPES buffer solution (50 mM) with 10 mM NaCl at pH 7.4 (25 °C).

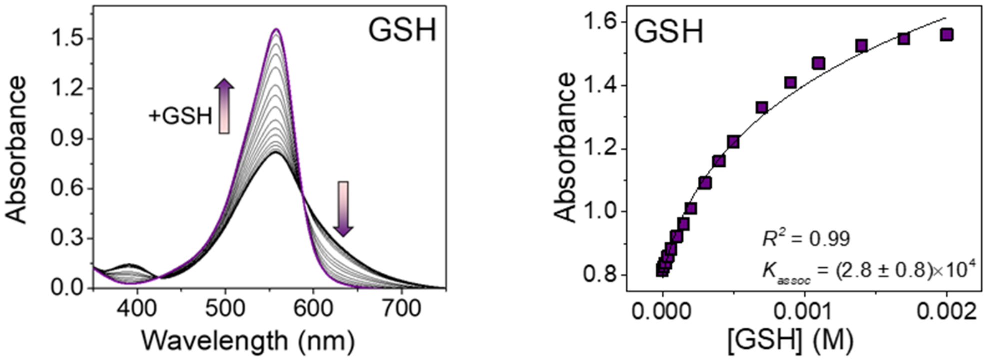

Supplementary Figure 18. UV–Vis spectra of the BPR (40 μM) – Zn^{2+} (40 μM) complex upon the addition of GSH (0–2.0 mM) in a HEPES buffer solution (50 mM) with 10 mM NaCl at pH 7.4 (25 °C).

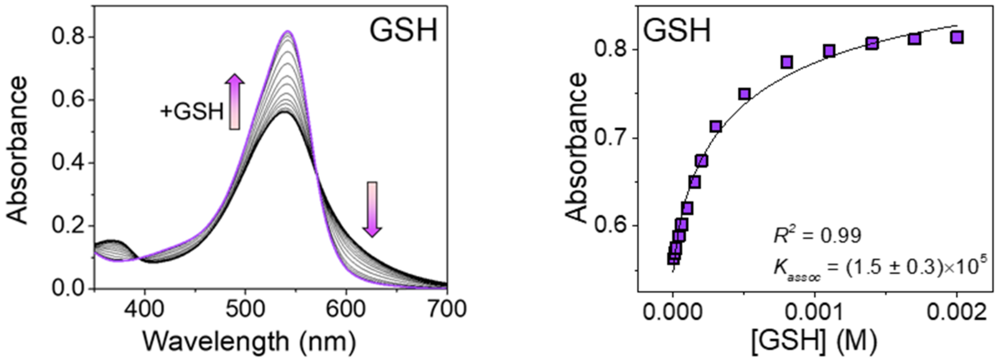

Supplementary Figure 19. UV–Vis spectra of the PR (40 μM) – Zn^{2+} (40 μM) complex upon the addition of GSH (0–2.0 mM) in a HEPES buffer solution (50 mM) with 10 mM NaCl at pH 7.4 (25 °C).

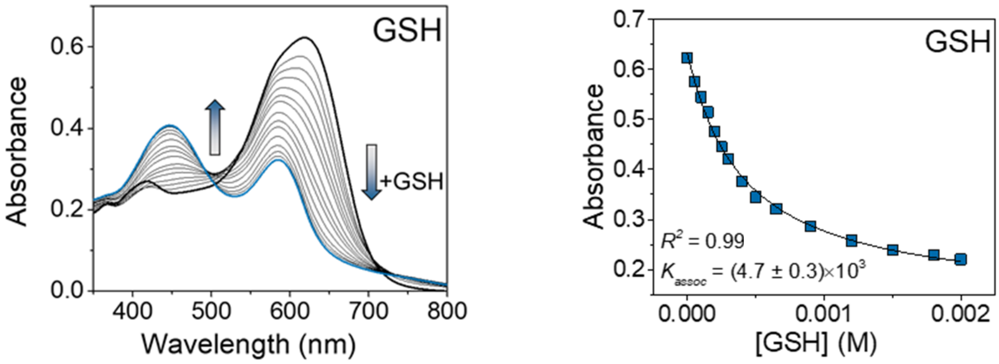

Supplementary Figure 20. UV–Vis spectra of the PV (40 μM) – Zn^{2+} (200 μM) complex upon the addition of GSH (0–2.0 mM) in a HEPES buffer solution (50 mM) with 10 mM NaCl at pH 7.4 (25 °C).

The association constants for the SCAAs with the Zn^{2+} ion were calculated by the titration isotherms according to a nonlinear regression fitting model, and the stoichiometry of indicator–analyte complex during the association constants calculation was fit to 1:1 modeled (Hargrove et al., 2010) by eqs. (1) and (2)

$$[\mathrm{H}]_t = [\mathrm{H}] + \frac{K_{assoc}[\mathrm{H}]}{1+K_{assoc}[\mathrm{H}]}[\mathrm{G}]_t + \frac{K_I[\mathrm{H}]}{1+K_I[\mathrm{H}]}[\mathrm{I}]_t \qquad (1)$$

$$A = \frac{[\mathrm{I}]_t}{1+K_I[\mathrm{H}]}(\varepsilon_I b + \varepsilon_{HI} b K_I[\mathrm{H}]) \qquad (2)$$

where $[\mathrm{I}]_t$ is the concentration of the catechol dye, $[\mathrm{H}]_t$ is the concentration of Zn^{2+} ion, $[\mathrm{G}]_t$is the concentration of SCAAs, and K_{assoc} and K_I refer to the association constants of the Zn^{2+}–SCAAs and Zn^{2+}–catechol dyes, respectively. [H] demonstrates the concentration of the unknown concentration of the Zn^{2+} ion, which could be calculated by K_{assoc} and K_I and by the experimentally obtained values including $[\mathrm{G}]_t$, $[\mathrm{H}]_t$ and $[\mathrm{I}]_t$. Moreover, in eq. (2), A represent to the SCAA–concentration–dependent absorbance, b donates the cuvette thickness, and ε_I and ε_{HI} mean the molar absorption coefficients of the catechol dye and the complex of the dye–Zn^{2+}, respectively.

2. Results of selectivity test

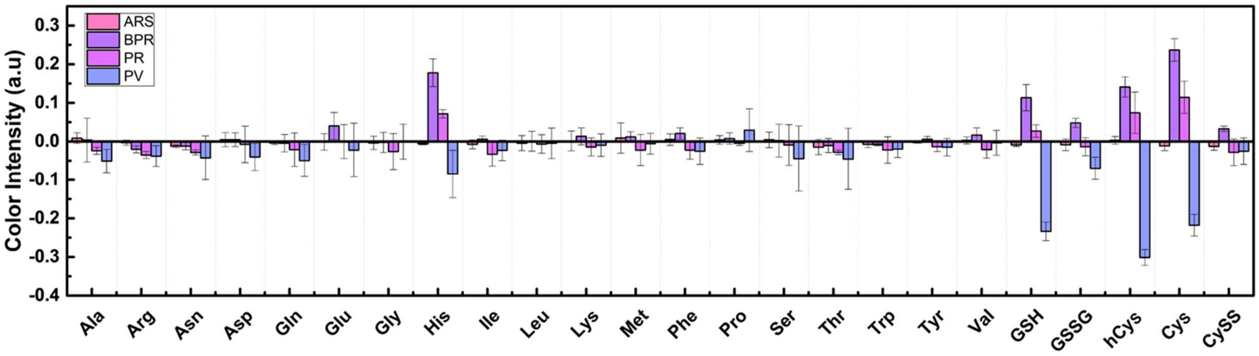

Supplementary Figure 21. Response profile of the catechol dye–Zn^{2+} upon addition of 24 analytes at 1.0 mM. Bar graphs were prepared from the varieties in the maximum absorption at 615 nm for PV, 557 nm for BPR, 543 nm for PR, and 515 nm for ARS, respectively.

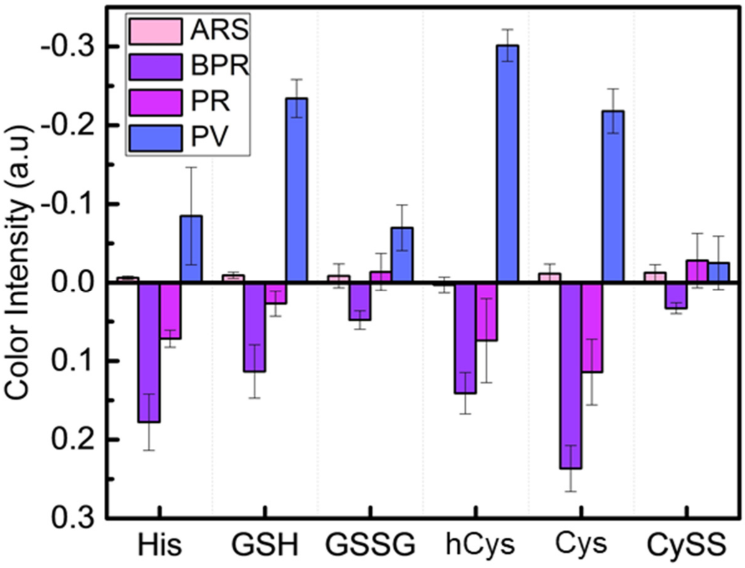

Supplementary Figure 22. Response profile of the catechol dye–Zn^{2+} upon addition of 6 analytes (5 SACCs and His) at 1.0 mM. Bar graphs were prepared from the varieties in the maximum absorption at 615 nm for PV, 557 nm for BPR, 543 nm for PR, and 515 nm for ARS, respectively.

3. Results of quantitative analysis

Supplementary Table 1. The jackknifed classification matrix of the qualitative assay.

Jackknifed Classification Matrix							
	Blank	Cys	GSH	GSSG	His	hCys	%correct
Blank	6	0	0	0	0	0	100
Cys	0	6	0	0	0	0	100
GSH	0	0	6	0	0	0	100
GSSG	0	0	0	6	0	0	100
His	0	0	0	0	6	0	100
hCys	0	0	0	0	0	6	100
Total	6	6	6	6	6	6	100

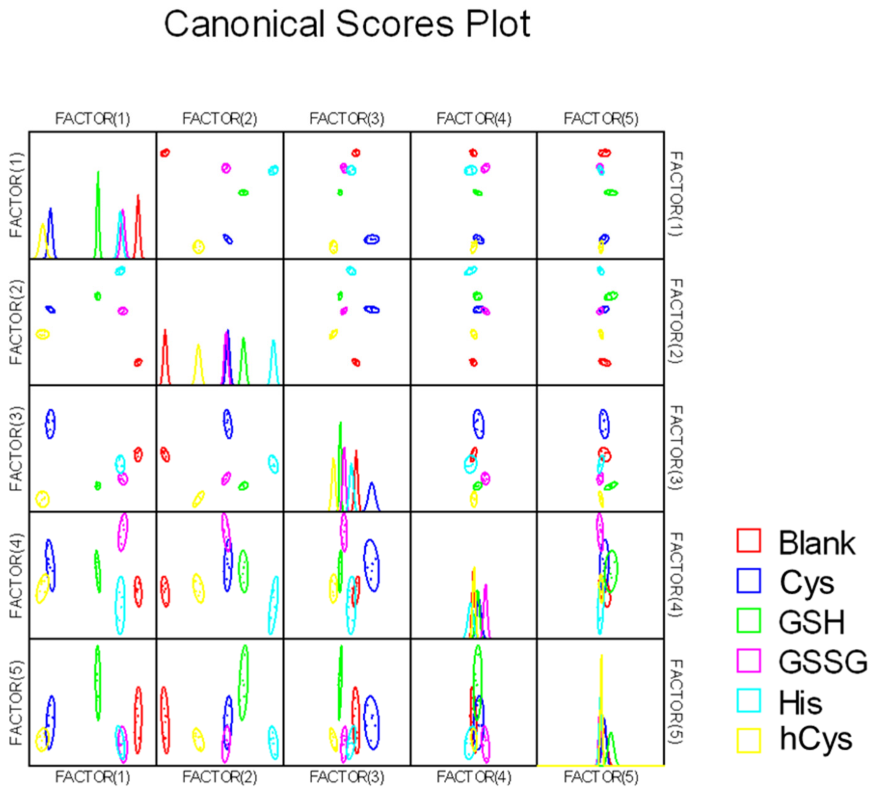

Supplementary Figure 23. The canonical score plots of the qualitative assay.

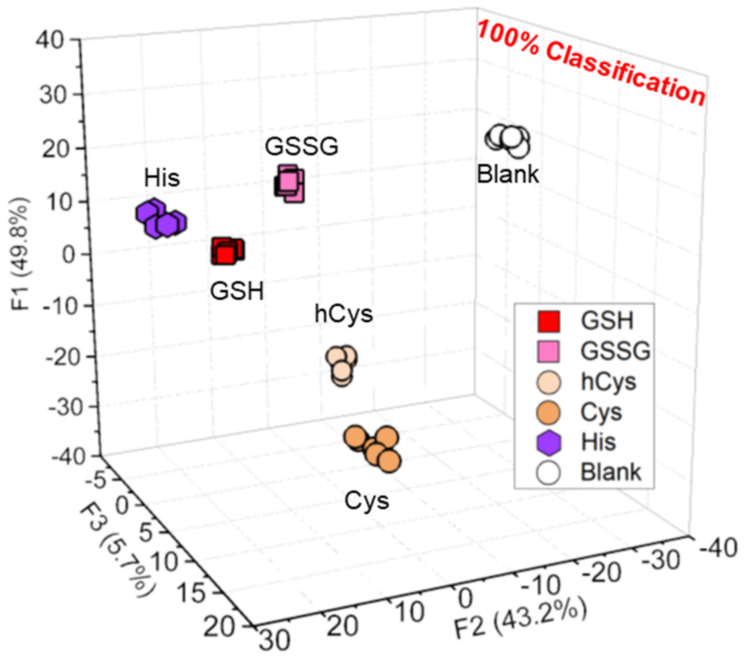

Supplementary Figure 24. LDA canonical score plots for the response pattern of the colorimetric chemosensor array to five analytes (plus a control dataset) (1.0 mM) in a HEPES buffer (50 mM) with 10 mM NaCl at pH 7.4 (25 °C). The measurements were repeated six times for each analyte. 100% correct classification of cross–validation was determined. The responses of all four dye–Zn^{2+} complexes were used.

4. Results of semiquantitative analysis

Supplementary Table 2. The jackknifed classification matrix of the semiqualitative assay for Cys–CySS.

Jackknifed Classification Matrix								
	0 µM Cys – 500 µM CySS	100 µM Cys – 400 µM CySS	150 µM Cys – 350 µM CySS	200 µM Cys – 300 µM CySS	250 µM Cys – 250 µM CySS	300 µM Cys – 200 µM CySS	400 µM Cys – 100 µM CySS	%correct
0 µM Cys – 500 µM CySS	18	0	0	0	0	0	0	100
100 µM Cys – 400 µM CySS	0	18	0	0	0	0	0	100
150 µM Cys – 350 µM CySS	0	0	18	0	0	0	0	100
200 µM Cys – 300 µM CySS	0	0	0	18	0	0	0	100
250 µM Cys – 250 µM CySS	0	0	0	0	18	0	0	100
300 µM Cys – 200 µM CySS	0	0	0	0	0	18	0	100
400 µM Cys – 100 µM CySS	0	0	0	0	0	0	18	100
Total	18	18	18	18	18	18	18	100

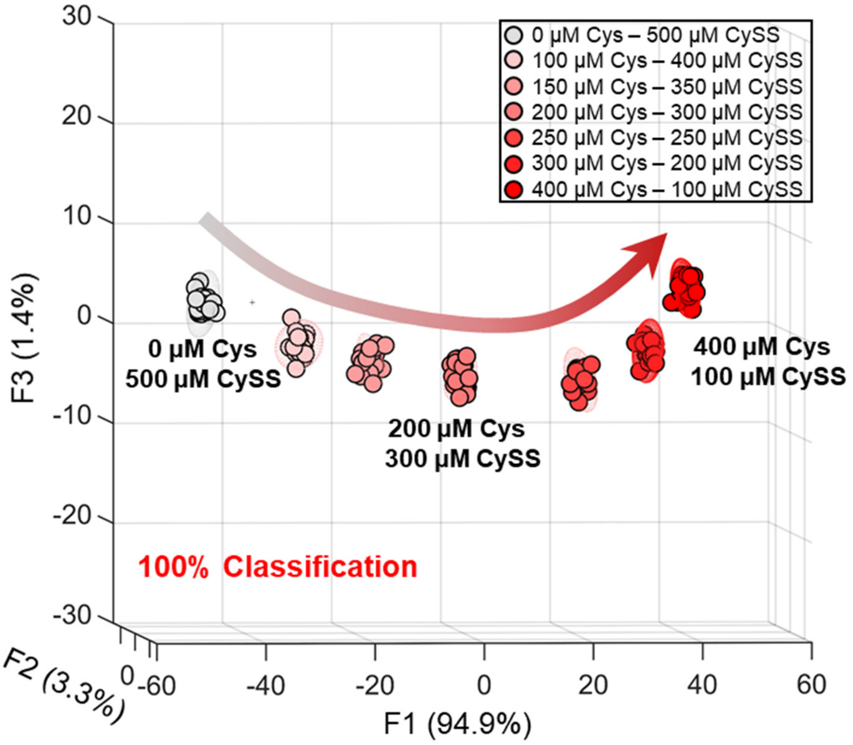

Supplementary Figure 25. Semiquantitative LDA result for various molar ratios of Cys and CySS. For each concentration, measurements were performed eighteen times, resulting in 100% successful classification. The confidence ellipsoids indicate 95% confidence.

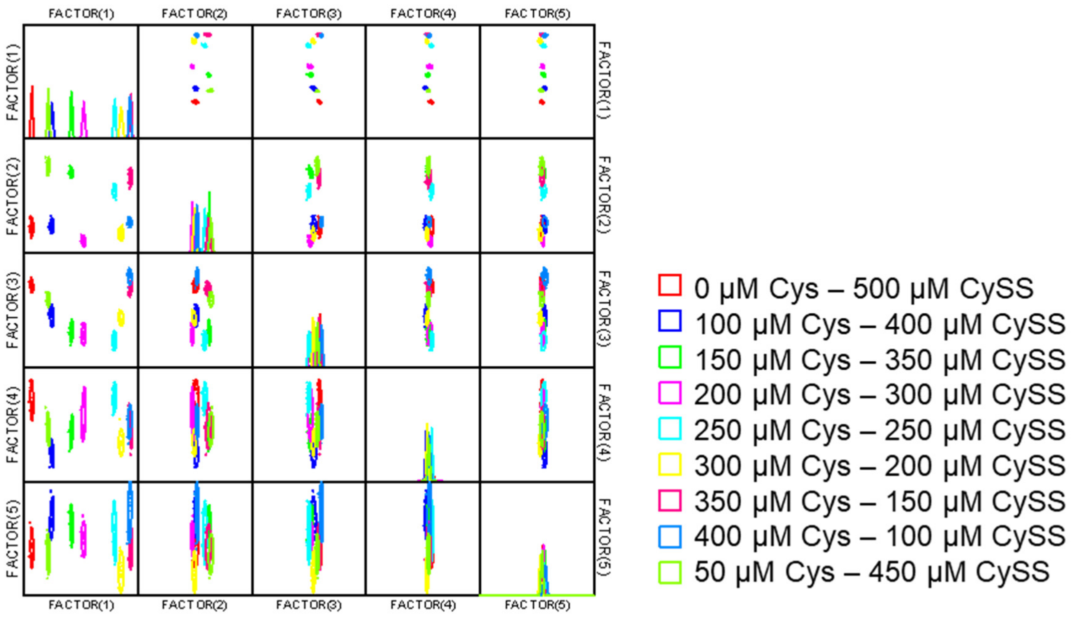

Supplementary Figure 26. The canonical score plots of the semiqualitative assay.

Supplementary Table 3. The jackknifed classification matrix of the semiqualitative assay for GSH–GSSG.

Jackknifed Classification Matrix							
	0 µM GSH – 900 µM GSSG	200 µM GSH – 800 µM GSSG	600 µM GSH – 600 µM GSSG	1000 µM GSH – 400 µM GSSG	1400 µM GSH – 200 µM GSSG	1800 µM GSH – 0 µM GSSG	%correct
0 µM GSH – 900 µM GSSG	18	0	0	0	0	0	100
200 µM GSH – 800 µM GSSG	0	18	0	0	0	0	100
600 µM GSH – 600 µM GSSG	0	0	18	0	0	0	100
1000 µM GSH – 400 µM GSSG	0	0	0	18	0	0	100
1400 µM GSH – 200 µM GSSG	0	0	0	0	18	0	100
1800 µM GSH – 0 µM GSSG	0	0	0	0	0	18	100
Total	18	18	18	18	18	18	100

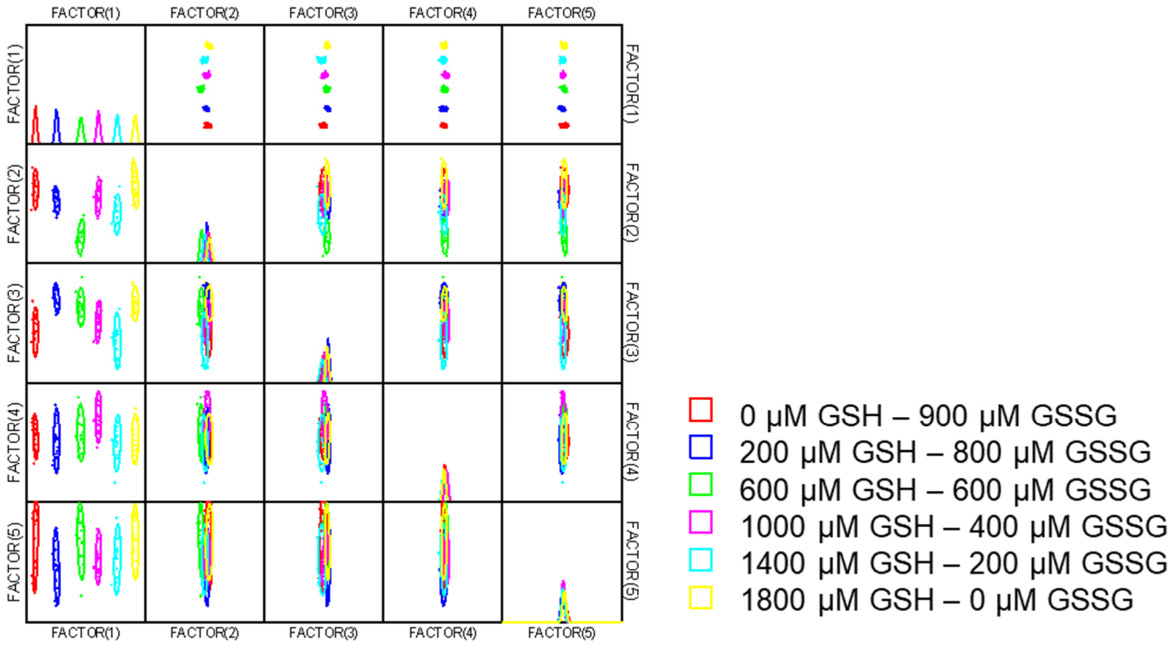

Supplementary Figure 27. The canonical score plots of the semiqualitative assay.

5. Results of quantitative analysis

Supplementary Table 4. Concentration conditions for the quantitative assay of the mixture of GSH and GSSG.

Cys (mM)	CySS (mM)
0.05	0.45
0.10	0.40
0.15	0.35
0.20	0.30
0.25	0.25
0.30	0.20
0.35	0.15
0.40	0.10
0.45	0.05

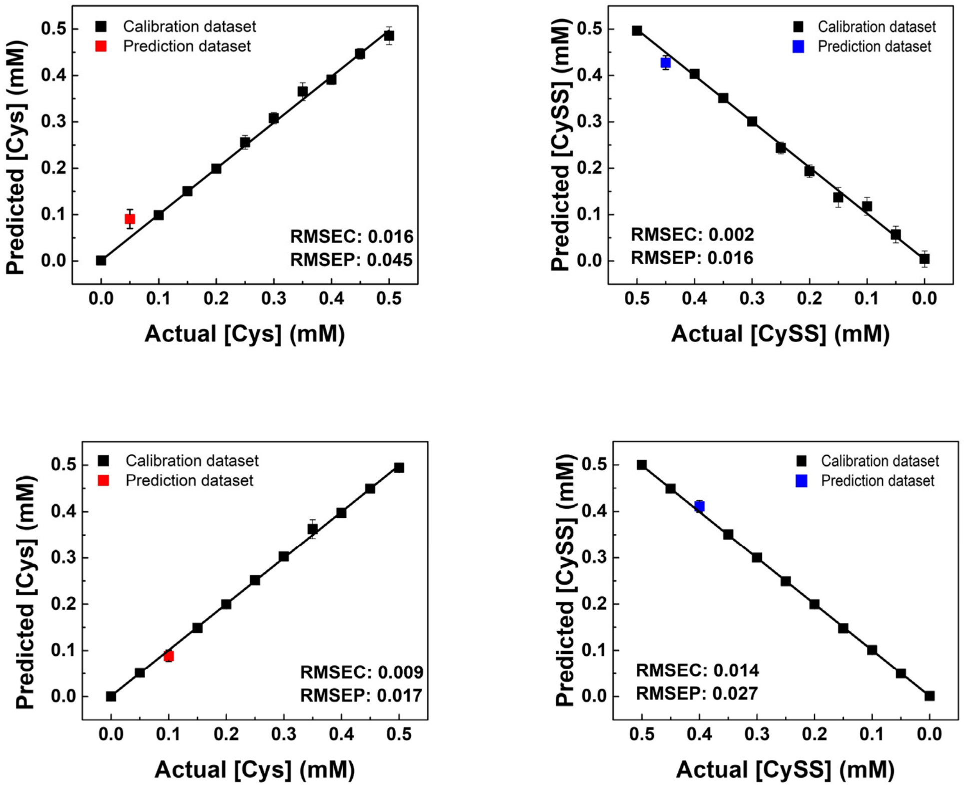

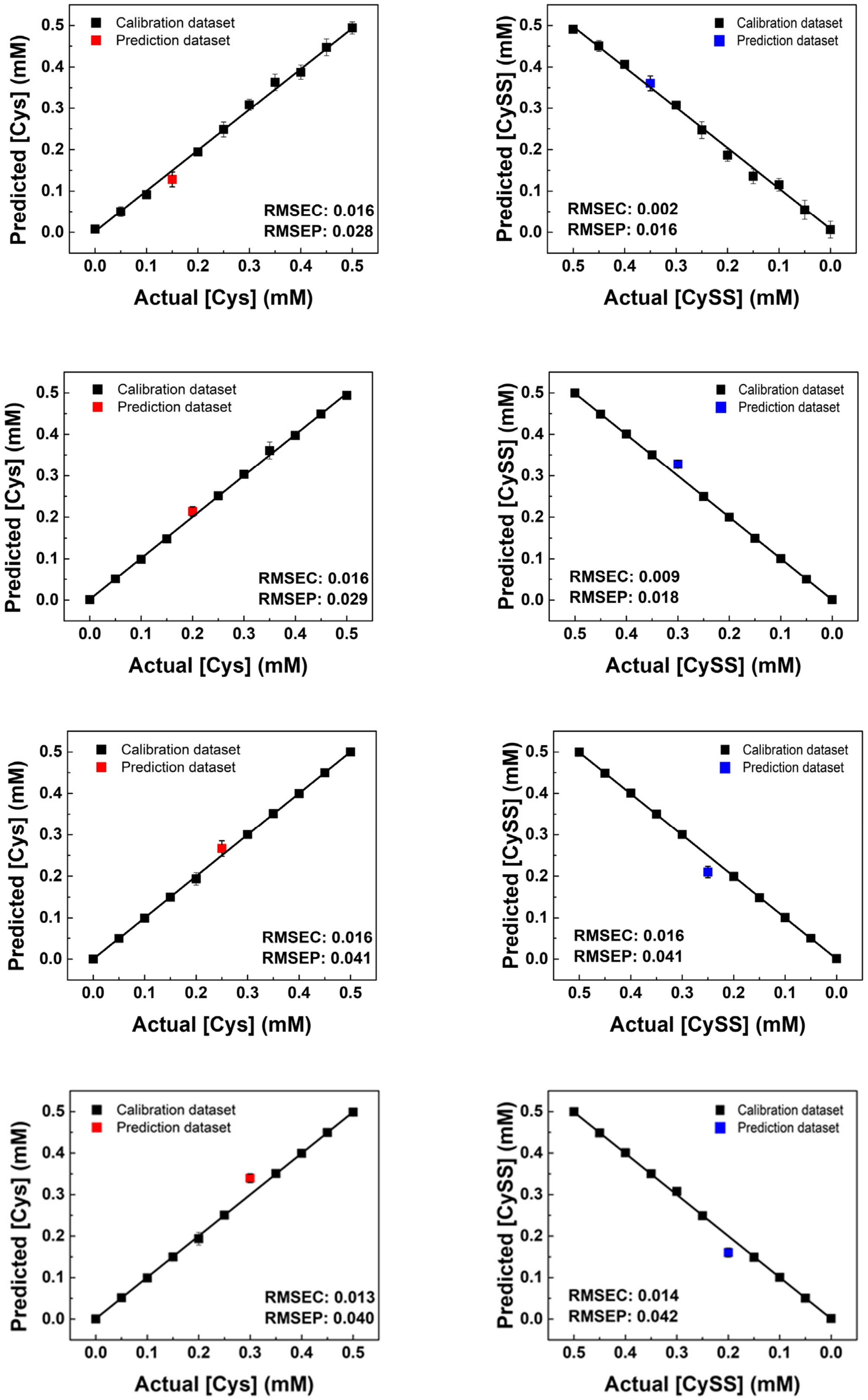
Calibration dataset
Prediction dataset
Predicted [Cys] (mM)
Actual [Cys] (mM)
RMSEC: 0.016
RMSEP: 0.028
Predicted [CySS] (mM)
Actual [CySS] (mM)
RMSEC: 0.002
RMSEP: 0.016
RMSEC: 0.016
RMSEP: 0.029
RMSEC: 0.009
RMSEP: 0.018
RMSEC: 0.016
RMSEP: 0.041
RMSEC: 0.016
RMSEP: 0.041
RMSEC: 0.013
RMSEP: 0.040
RMSEC: 0.014
RMSEP: 0.042

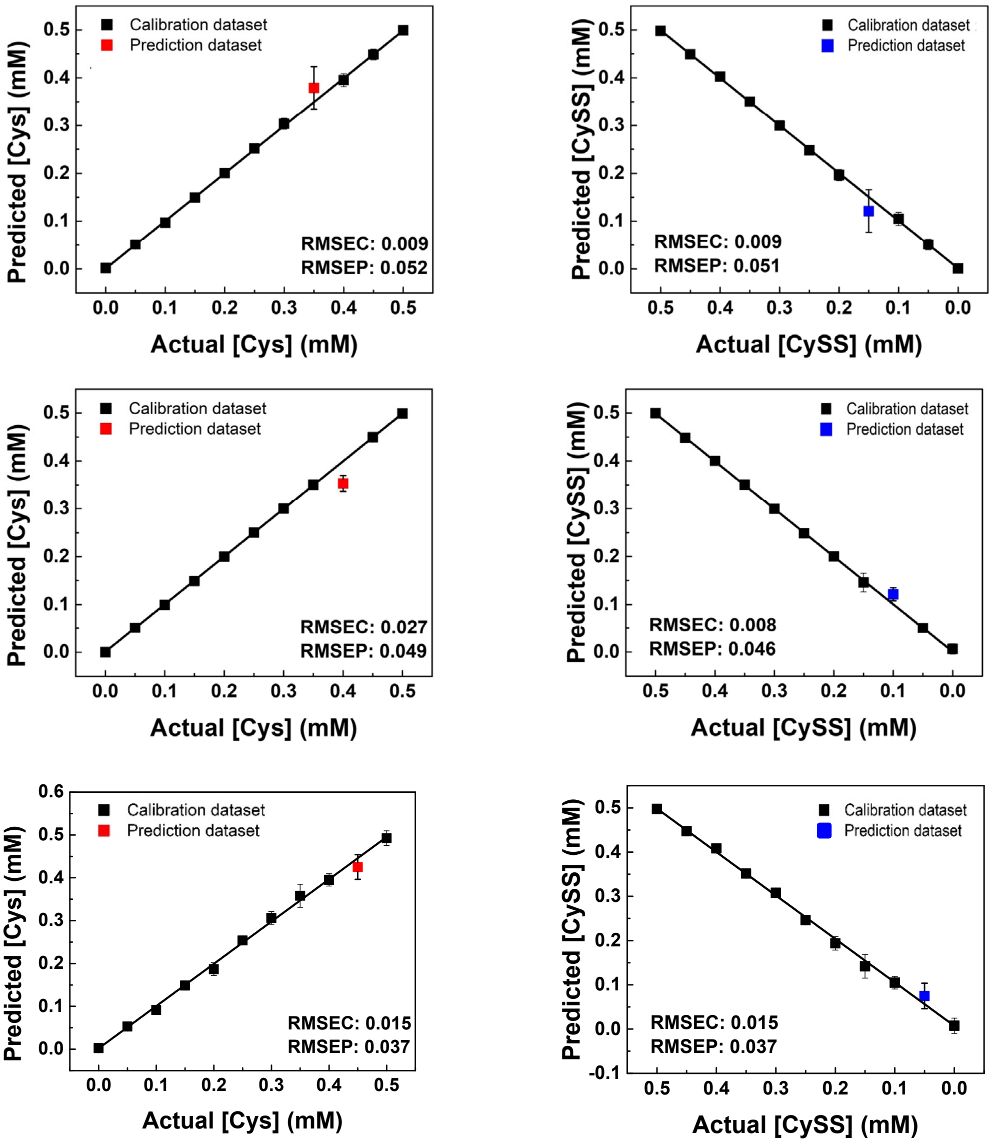

Supplementary Figure 28. SVM regression for quantitative analysis of the mixtures of Cys and CySS.

Supplementary Table 5. Concentration conditions for the quantitative assay of the mixture of GSH and GSSG.

GSH (mM)	GSSG (mM)
0.2	0.8
0.4	0.7
0.6	0.6
0.8	0.5
1.0	0.4
1.2	0.3
1.4	0.2
1.6	0.1

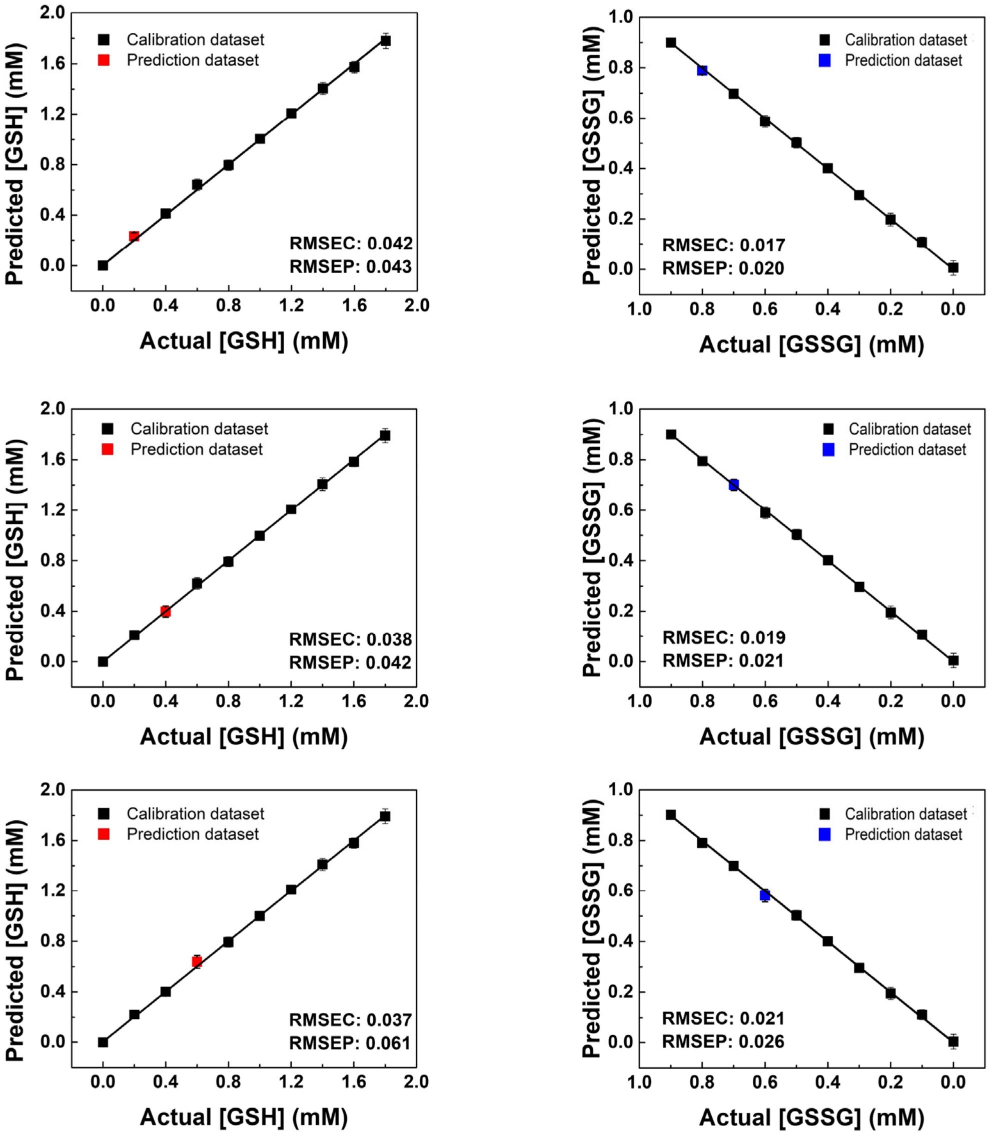

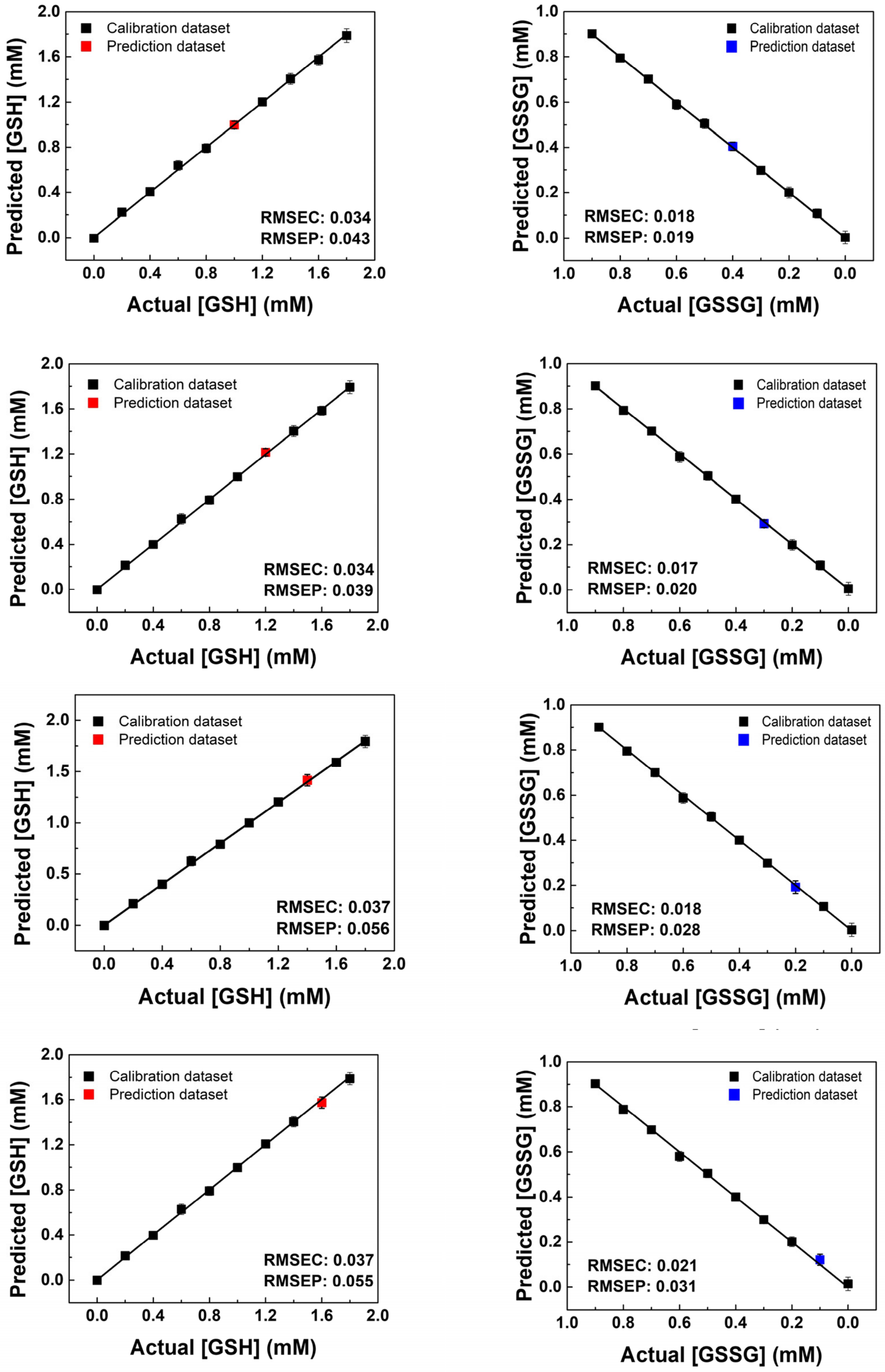

Supplementary Figure 29. SVM regression for quantitative analysis of the mixture of GSH and GSSG.

6. Real-sample analysis for sulfur–containing amino acids

Supplementary Table 6. The results of the spike test.

<table>
<tr><th rowspan="6">Ito En Tomato Juice</th><th>Added (μM)</th><th>Founded (μM)</th><th>Recovery (%)</th><th rowspan="6">Fresh Tomato</th><th>Added (μM)</th><th>Founded (μM)</th><th>Recovery (%)</th><th rowspan="6">Asahi Juice Welch's Grapefruit Juice</th><th>Added (μM)</th><th>Founded (μM)</th><th>Recovery (%)</th></tr>
<tr><td>0</td><td>201.0 ± 21.9</td><td>-</td><td rowspan="2">0</td><td rowspan="2">129.9 ± 18.4</td><td rowspan="2">-</td><td>0</td><td>250.7 ± 13.6</td><td>-</td></tr>
<tr><td>100</td><td>300.4 ± 14.0</td><td>99.4</td><td rowspan="2">100</td><td rowspan="2">344.2 ± 29.0</td><td rowspan="2">93.4</td></tr>
<tr><td>200</td><td>415.2 ± 21.4</td><td>107.1</td><td>100</td><td>221.1 ± 33.0</td><td>91.3</td></tr>
<tr><td>300</td><td>530.9 ± 39.2</td><td>110</td><td>300</td><td>346.0 ± 41.3</td><td>108</td><td rowspan="2">200</td><td rowspan="2">450.2 ± 22.9</td><td rowspan="2">99.7</td></tr>
<tr><td>400</td><td>600.1 ± 29.9</td><td>99.8</td><td>200</td><td>460.7 ± 5.5</td><td>110</td></tr>
</table>

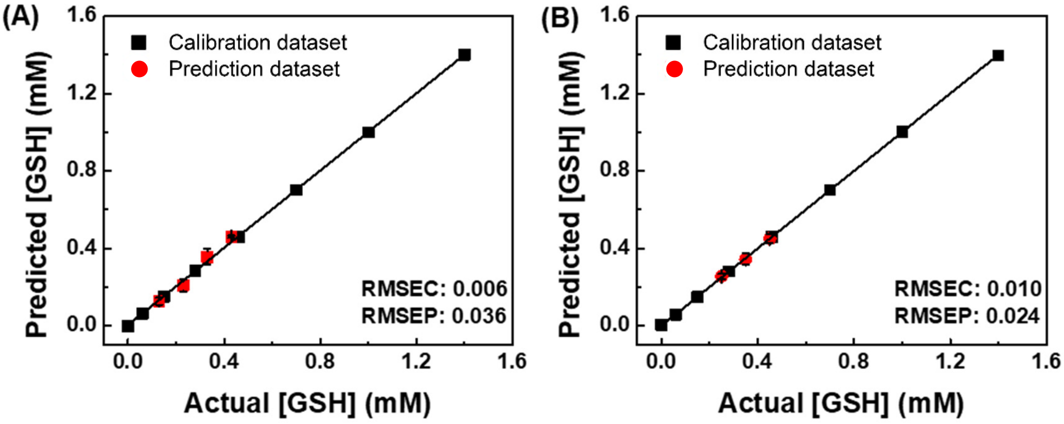

Supplementary Figure 30. SVM regression for real sample analysis of GSH in (A) a tomato sample and (B) Asahi Juice Welch's Grapefruit Juice. The RMSEC and RMSEP values (shown as insets) represent the accuracy of the constructed model and the prediction.

7. HPLC analysis

Sample Preparation

Freshly made tomato juices were firstly centrifuged at 14,000 rpm for 10 mins. The supernatant (500 μL) was then diluted for 1 time using 50 mM citrate buffer at pH 5.0. For oxidization process, H_2O_2 (10 μL, 30%) was added in the diluted solution under strongly vortexed conditions, which stood for 10 min at room temperature. On the other hand, the standard GSH solutions ([GSH] = 10, 30, 50, 70, 100 μM) were prepared by dissolving GSH in 50 mM citrate buffer at pH 5.0.

Sample Labeling

The prepared sample solutions (100μL) were mixed with a methanol solution (50 μL) of pBQ (400 μM) to form N^5-(1-((carboxymethyl)amino)-3-((2,5-dihydroxyphenyl)thio)-1-oxopropan-2-yl)glutamine (GSH-HQ) as previously reported (Tirelli, et al., 2010). After vortexing for 1 min, a citrate buffer solution (0.3 M at pH 3.5, 500 μL) containing 3-MPA (500 μM) was added to the above solutions for reacting with the excess amount of pBQ. Subsequently, the solutions were filtered using a membrane filter (0.22 μm, AS ONE Corp. Syringe Filter (Hydrophilic) 004022NL-SEPTFE).

HPLC Separation for GSH-HQ

The reverse-phase HPLC separation of GSH-HQ was performed using a Waters XBridge BEH Phenyl Column, 130 Å, 3.5 µm, 4.6 mm × 150 mm. The mobile phase was trifluoroacetic acid/Milli-Q water (0.05% v/v) and methanol, with the gradient flowing speed at 1.0 mL/min. The concentration of methanol increased from 5% to 13.7% in 10 min, which then jumped and kept at 25% until directly dropped back to 5% at 18 min and end at 22 min. HPLC settings were as follows: a channel of UV detector: 303 nm; a column oven: 40 °C; an injection volume: 25 µL. Chromatographic data were acquired and processed using Shimadzu LabSolutions (Shimadzu, Japan).

LC-MS Analysis

The HPLC (Shimadzu LC-2010CHT) and electrospray ionization-mass spectrometry (ESI-MS, Shimadzu LCMS-2020) were controlled by the LabSolutions. The settings for the ESI-MS were as follows: analysis mode, positive scan; interface potential, 5.0 kV; nebulizer gas (N_2) flow rate, 1.5 L/min; capillary temperature, 275 °C; Qarray RF voltage, 30 V; and range, 50-800 m/z. Mass data were acquired and processed using Shimadzu LabSolutions.

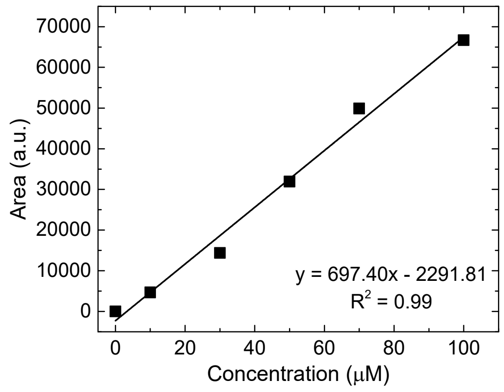

Supplementary Figure 31. HPLC calibration curve obtained from 0, 10, 30, 50, 70, and 100 µM GSH standard solution.

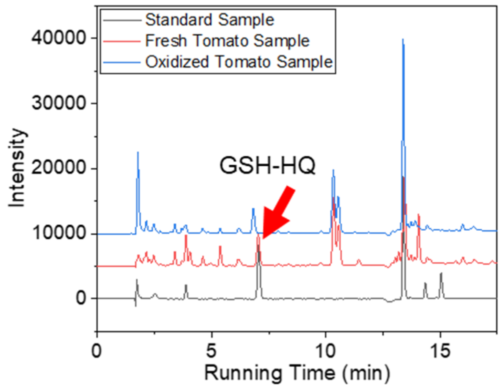

Supplementary Figure 32. HPLC chromatographic chart of the GSH standard solution (100 µM), the fresh tomato juice solution, and the oxidized tomato juice solution. In the oxidized tomato solution, the GSH-HQ peak could not be observed, supported by ESI-MS.

Supplementary Table 7. The HPLC result of the GSH concentration in diluted fresh tomato juices

Test Number	GSH (μM)
1	67.9
2	61.8
3	60.8
Avg.	63.5

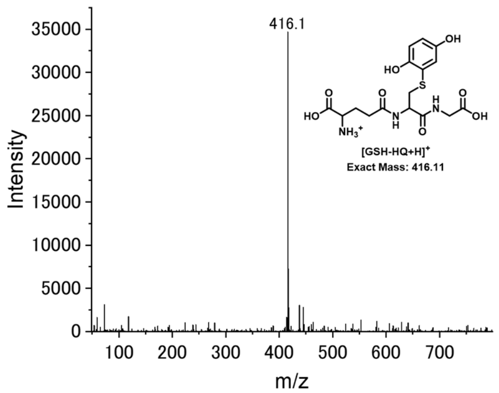

Supplementary Figure 33. ESI-MS spectrum of GSH-HQ in the fresh tomato sample.

8. LDA for the freshness monitoring of tomato

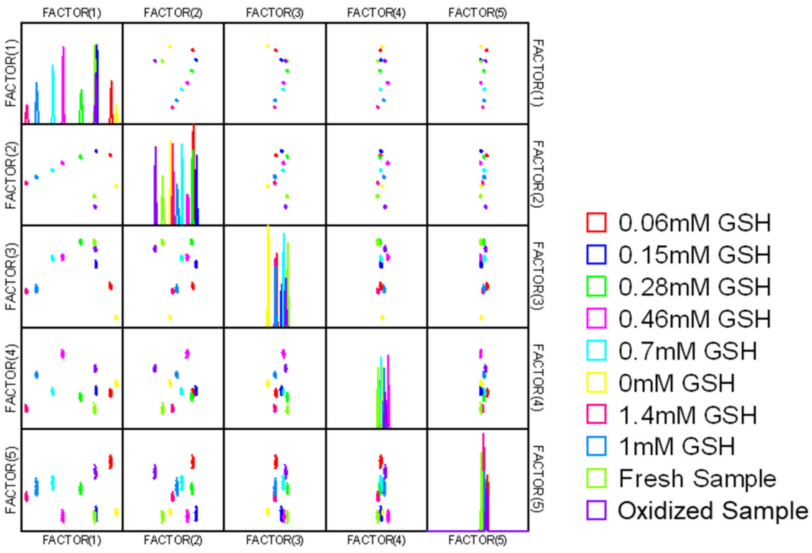

Supplementary Figure 34. The canonical score plots of the qualitative assay including the tomato samples.

Supplementary Table 8. The jackknifed classification matrix of the real sample test.

Jackknifed Classification Matrix											
	0.06mM GSH	0.15mM GSH	0.28mM GSH	0.46mM GSH	0.7mM GSH	0mM GSH	1.4mM GSH	1mM GSH	Fresh Sample	Oxidized Sample	%correct
0.06mM GSH	20	0	0	0	0	0	0	0	0	0	100
0.15mM GSH	0	20	0	0	0	0	0	0	0	0	100
0.28mM GSH	0	0	20	0	0	0	0	0	0	0	100
0.46mM GSH	0	0	0	20	0	0	0	0	0	0	100
0.7mM GSH	0	0	0	0	20	0	0	0	0	0	100
0mM GSH	0	0	0	0	0	20	0	0	0	0	100
1.4mM GSH	0	0	0	0	0	0	20	0	0	0	100
1mM GSH	0	0	0	0	0	0	0	20	0	0	100
Fresh Sample	0	0	0	0	0	0	0	0	20	0	100
Old Sample	0	0	0	0	0	0	0	0	0	20	100
Total	20	20	20	20	20	20	20	20	20	20	100

References

Hargrove, A.E., Zhong, Z., Sessler, J.L., and Anslyn, E.V. (2010). Algorithms for the determination of binding constants and enantiomeric excess in complex host : guest equilibria using optical measurements. *New J. Chem.* 34**,** 348–354. doi: 10.1039/B9NJ00498J

Tirelli, A., Fracassetti, D., and Noni, I.D. (2010). Determination of Reduced Cysteine in Oenological Cell Wall Fractions of Saccharomyces cerevisiae. *J. Agric. Food Chem*. 2010, 58, 4565–4570. doi:10.1021/jf904047u
